# Charge Transfer and Recombination Pathways through Fullerene Guests in Porphyrin-Based MOFs

**DOI:** 10.1021/acs.jpcc.5c00161

**Published:** 2025-04-23

**Authors:** Alison Arissa, Thomas Rose, Noémi Leick, Stefan Grimme, Justin C. Johnson, Jenny V. Lockard

**Affiliations:** † Department of Chemistry, 67206 Rutgers University-Newark, Newark, New Jersey 07102, United States; ‡ Mulliken Center for Theoretical Chemistry, Clausius-Institut für Physikalische und Theoretische Chemie, Rheinische Friedrich-Wilhelms Universität Bonn, Bonn 53115, Germany; § 53405 National Renewable Energy Laboratory, 15013 Denver West Parkway, Golden, Colorado 80401, United States

## Abstract

Porphyrin-based metal–organic frameworks (MOFs) offer a unique platform for building porous donor–acceptor networks that exhibit long-lived charge separation and transport upon incorporation of electron acceptor guest species. Here, porphyrin-based MOFs, PCN-222­(H_2_) and PCN-222­(Zn), synthesized as nanoparticle suspensions, are successfully infiltrated with fullerene acceptor molecules, C_60_ or PC_61_BM, in both polar and nonpolar solvent environments. The location and relative binding strength of these guest species are evaluated through a combination of N_2_ physisorption measurements, photoluminescence quenching, and UV–vis absorption titration experiments. Semiempirical tight binding calculations are used to screen potential locations of the fullerene guest within the MOF pores, and hybrid density functional theory (DFT)-computed interaction energies confirm the energetically favorable positions. The fundamental photophysics of these donor–acceptor host–guest combinations are probed using ultrafast transient absorption spectroscopy. Sub-picosecond electron transfer involving initial exciplex population is observed, with slow charge recombination lifetimes on the order of τ ∼1 ns for all systems in both dimethylformamide and 1,4-dioxane. Charge recombination occurs through population of fullerene and/or framework porphyrin triplet states depending on the porphyrin metalation status. The photophysics of the fullerene-loaded MOFs are discussed in the context of relevant porphyrin–fullerene donor–acceptor molecules to highlight the unique role of the framework environment in dictating photoinduced electron transfer and decay pathways.

## Graphical abstract

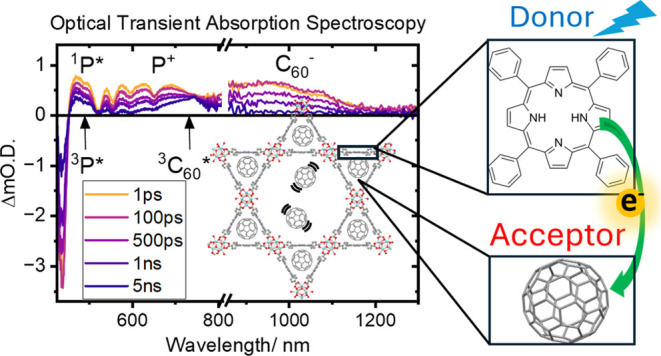

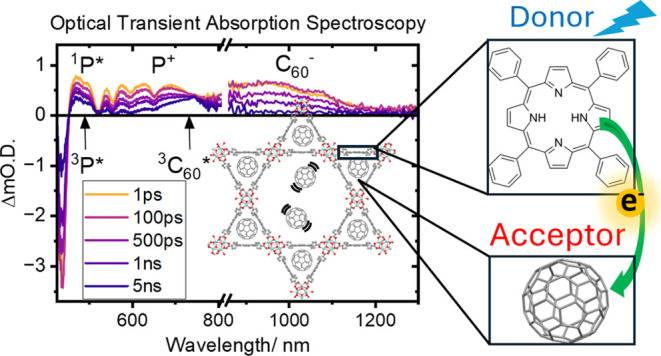

## Introduction

Metal–organic frameworks (MOFs) present both technological promise in a wide array of applications and a fruitful platform for fundamental studies of host–guest chemistry, electron transfer, and photophysics in three-dimensional (3D) coordination space.^1–6^ Composed of metal ions or clusters connected through coordination bonds with organic or organometallic linkers, MOFs self-assemble under controlled solvothermal conditions to yield a variety of microporous environments and precise chemical tunability not found in other solid-state hybrid materials.^7,8^ The retention of crystallinity upon solvent removal or exchange renders the porous frameworks accessible to other potential guest species during subsequent postsynthetic modification (PSM) treatment. This provides another handle to further alter the framework composition and electronic structure. The resulting diversity in chemical makeup and tunable, permanent porosity make MOFs attractive candidates for potential adsorption-based applications. While initial targets focused on gas separation and storage,^9–12^ and catalysis,^13–15^ more recent efforts to impart and study electron transfer and transport properties in these materials pave the way for applications that rely on MOF conductivity and/or long-lived charge separation such as resistive sensors, electrochromic devices, and electro- or photocatalysis.^16–26^ In one approach, introducing redox-active guest species via PSM can yield MOF host–guest donor–acceptor (D–A) systems with the desired charge transfer (CT) and transport properties. Leveraging established molecular D–A combinations, focusing solely here on archetypal electron-rich porphyrins and electron-deficient fullerenes, organized D–A arrangements within the porous structures can be achieved through host–guest interaction. Supramolecular porphyrin–fullerene D–A complexes, which have been studied extensively,^27–30^ typically rely on self-assembly methods to build extended arrays to reach longer length scales needed for device fabrication.^31,32^ In MOF-based systems, the introduction of fullerene acceptor guest molecules via postsynthetic modification into preformed MOF structures containing porphyrin donor linkers can promote these D–A interactions upon confinement within the porous structure while avoiding additional self-assembly steps. The ideal framework for most applications, and in particular photo­(electro)­catalysis, would promote selective infiltration of these fullerene guests to foster the D–A host–guest interactions yet possess a pore structure that remains accessible to other guest molecules. PCN-222 is a MOF structure predicted to exhibit such behavior.

The PCN-222 framework contains nodes of Zr_6_ clusters connected by tetracarboxyphenylporphyrin linkers to form a 3D architecture with two types of one-dimensional (1D) channel pores that are considerably different in size and shape.^33^ The smaller of the two channels is ideally suited for housing electron-deficient molecules such as C_60_ and other fullerene derivatives. Preferential confinement within these triangular pores would likely facilitate their D–A interactions with the host framework linkers, triggering directional charge transport. The much larger hexagonal porous channels, however, would not likely have this confinement effect on fullerene, leaving them effectively available for other guest species. The isostructural framework, NU-1000 (with tetracarboxyphenyl pyrene in place of the porphyrin linkers)^34^ provides precedent for this host–guest arrangement and D–A behavior. Introducing metal­(IV) bis­(dicarbollide) complexes as electron-deficient guests to this framework leads to preferential confinement within the smaller channels, and the composite host–guest material shows increased electrical conductivity.^35^ Furthermore, photoconductive behavior is predicted for this guest molecule as well as C_60_ incorporated in metal-free or zinc porphyrin versions of PCN-222.^36^ The photophysics and electrochemistry of similar porphyrin units, along with the precedent for noncovalent D–A interaction with electron acceptor molecules in cage complexes and other frameworks,^29,30,37–39^ suggests their likely participation as electron donors upon visible light irradiation in these PCN-222 host–guest systems. An additional advantage of MOFs with multiple pore types is the unique chemical environments that they might exhibit due to steric effects, mass transport limitations, or pore surface chemistry that is distinct in different pores.

In this investigation, we examine a series of PCN-222 frameworks loaded with fullerene guest molecules to experimentally confirm the predicted confinement within the porous structure and accompanying D–A interaction with the porphyrin linkers. While charge transport is expected to be an important property of these materials for some applications, this study focuses on understanding the initial photoinduced CT process and associated decay pathway of these systems. C_60_ or fullerene derivative, [6,6]­phenyl-C_61_-butyric acid methyl ester (PC_61_BM), guest species are incorporated in either the metal-free or zinc porphyrin version of this MOF, PCN-222­(H_2_,Zn), to probe the steric effects of guest functionalization and influence of porphyrin metalation status on the host–guest cofacial binding strength and resulting D–A excited state decay pathways. Both *N*′,*N*-dimethylformamide (DMF) and 1,4-dioxane solvent environments are employed to determine the influence of the solvent polarity on these excited state dynamics. Steady state electronic spectroscopy combined with N_2_ gas physisorption measurements are used to establish the location and relative binding strength of the fullerene guests within the frameworks, while ultrafast optical transient absorption (TA) spectroscopy studies reveal their excited state landscapes and associated dynamics. Optical spectroscopic measurements of MOF materials, in general, can be challenging because of light scattering interference. Here, we use a modified MOF synthesis method that generates extremely stable nanoparticle suspensions of PCN-222 to allow for in-depth steady state and time-resolved optical spectroscopy characterization. The photophysics of the porphyrin and fullerene components in other D–A contexts are well established,^40–44^ and therefore provide a useful benchmark to help interpret the TA results of the MOF host–guest systems and the observed trends. By tracking the spectral signatures of the charge transfer and triplet excited states following porphyrin photoexcitation, we observe subtle differences in both the rate of electron transfer and the recombination pathways depending on the fullerene guest/porphyrin linker combination. Furthermore, global fitting and target analysis of these TA data reveal that exciplex formation likely precedes the CT state population along the decay path. These results are discussed in the context of related D–A molecular systems and their established photoinduced electron transfer pathways. The comparisons highlight how the pore structure of the MOF uniquely promotes confinement of the acceptor species along the rigid framework channels, which in turn influences the charge transfer and recombination behavior toward specific triplet state populations.

## Experimental Methods

### Materials

*meso-*Tetracarboxyphenylporphyrin (TCPP), tetraphenylporphyrin (TPP), and zinc tetraphenylporphyrin (ZnTPP) were synthesized using literature precedent.^45–47^ Zirconyl chloride octahydrate (99% purity) was purchased from Sigma-Aldrich. *N*′,*N*-Dimethylformamide (DMF), toluene, and 1,2-orthodichlorobenzene (ODCB) were purchased from Millipore Sigma. Dichloroacetic acid (DCA) (97%) was purchased from TCI Chemicals. Buckminsterfullerene (C_60_) (99.9% purity) was obtained from Thermo Fisher Scientific, and [6,6]-phenyl-C_61_-butyric acid methyl ester (PC_61_BM) was obtained from Nano-C.

### Synthesis

PCN-222 nanoparticles were synthesized following literature precedent^48^ with some procedure modifications. 24 mg of TCPP was added to a 100 mL pressure vessel containing 60 mL of DMF and ultrasonicated until dissolved. 180 mg of ZrOCl_2_·8H_2_O and 1 mL of DCA were subsequently added, and the reaction flask was ultrasonicated for another 10 min. Finally, the reaction flask was placed in a 135 °C oven for 24 h. After cooling to room temperature, solutions were centrifuged in 40 mL of DMF at 10,000 rpm for 10 min. Subsequently, the supernatant was decanted, 40 mL of fresh DMF was added, and the nanoparticles were resuspended through ultrasonication for 30 min. This process was repeated two times. To ensure the removal of ZrOCl_2_·8H_2_O and TCPP starting material, the nanoparticles were resuspended in 20 mL of a 1 M HCl/DMF mixture using ultrasonication and left in a 120 °C oven for 18 h. Next, 20 mL of DMF was added to the suspension, which was then centrifuged for 10 min at 10,000 rpm. Finally, the supernatant was decanted and 40 mL of DMF was added to resuspend the nanoparticles. PCN-222­(Zn) nanoparticles were synthesized by postmetalation of PCN-222­(H_2_) using a previously reported procedure^49^ with slight modification. 100 mg of ZnCl_2_ was added to 40 mg of PCN-222­(H_2_) suspended in 20 mL DMF. The mixture was heated to 90 °C for 24 h. After cooling to room temperature, the PCN-222­(Zn) suspension was centrifuged at 10,000 rpm for 10 min, decanted, and resuspended in 20 mL 0.5 M HCl/DMF. Subsequently, the 0.5 M HCl/DMF suspension was centrifuged at 10,000 rpm for 10 min, decanted, and resuspended in 40 mL fresh DMF. After the centrifugation step in fresh DMF was repeated two more times, the PCN-222­(Zn) nanoparticles were resuspended a final time in DMF. To generate these MOF suspensions in 1,4-dioxane, solvent exchange was performed by centrifuging the DMF nanoparticle suspension at 10,000 rpm for 10 min followed by decantation and resuspension of the solid in 40 mL of 1,4-dioxane. This process was repeated five times.

#### C_60_⊂PCN-222­(H_2_,Zn) and PC_61_BM⊂PCN-222­(H_2_,Zn)

For powder X-ray diffraction and N_2_ physisorption measurements, 40 mg of washed PCN-222­(H_2_) or PCN-222­(Zn) nanoparticles suspended in 10 mL of DMF were combined with 100 mg of C_60_ or PC_61_BM dissolved in 10 mL of ODCB and allowed to soak for 7 days. This 8:1 fullerene/porphyrin ratio PCN-222­(H_2_,Zn) nanoparticle suspension was then centrifuged at 10,000 rpm for 10 min. After the supernatant was decanted, the nanoparticles were subsequently washed and centrifuged in 40 mL of toluene two times to achieve solvent exchange and remove unbound C_60_. Following the final decantation step, the sample was allowed to air-dry overnight. For transient absorption spectroscopy, PCN-222­(H_2_) or PCN-222­(Zn) nanoparticle suspensions in ∼10 mL of DMF (or 1,4-dioxane) were concentration adjusted to achieve an optical density of ∼0.2 at 400 nm absorption wavelength (resulting porphyrin concentration is ∼6.7 × 10^–6^ M). Subsequently, a 200 μL aliquot of C_60_ in toluene was added to achieve a final molar ratio in a solution of 8:1 C_60_ guest to porphyrin linker. The samples were allowed to soak for 7 days.

### Characterization

The zinc content in PCN-222­(Zn) was evaluated using atomic absorption spectroscopy (AAS) (Thermo Scientific iCE 3500 with a Zn hollow cathode lamp). 10 mg of PCN-222­(Zn) was activated in a vacuum oven at 130 °C for 24 h. Then, 7 mg was added to 10 mL of saturated NaOH and allowed to digest overnight prior to the AAS measurement. PCN-222­(H_2_,Zn) nanoparticles were characterized by scanning electron microscopy (SEM) and dynamic light scattering (DLS). SEM images were captured on a Hitachi S-4800 field emission scanning electron microscope using a secondary electron detector with an accelerating voltage of 15 kV and a probe current of 10 mA. SEM samples were prepared on a fixed aluminum stub, carbon conductive tape, and 1 mm of iridium sputter treatment. DLS measurements of the PCN-222­(H_2_,Zn) nanoparticle suspensions in DMF were obtained using a Malvern Zetasizer Nano-ZS instrument equipped with a 4 mW, 633 nm He–Ne laser and an Avalanche photodiode detector at an angle of 173°. Powder X-ray diffraction (PXRD) measurements were completed on a Rigaku MiniFlex 6G from 2 to 60° 2θ running at 40 kV and 15 mA (600 W). UV–visible absorption spectra were collected on a Cary 5000 UV–vis spectrophotometer in a quartz cell with a 1 cm optical path. The fluorescence data were collected on a Horiba Fluorolog-3 spectrofluorometer using λ = 421 or 430 nm excitation wavelength for the metal-free porphyrin- and Zn porphyrin-containing frameworks, respectively. Emission lifetime measurements were collected using a Light Conversion Harpia ultrafast spectroscopy system equipped with time-correlated single-photon counting (TCSPC) capability. λ = 421 or 430 nm excitation pump pulses were generated using a Yb:KGW pumped femtosecond laser source run at 10 kHz with noncollinear optical parametric amplification.

### N_2_ Physisorption

The samples were degassed against vacuum (1 × 10^–5^ Torr) at room temperature for 15 h, heated to 120 °C in 30 min, and held at 120 °C for 2.5 h prior to surface area and pore size distribution measurements. This procedure was performed in a home-built temperature-programmed desorption setup equipped with a residual gas analyzer (RGA) able to monitor mass-to-charge ratios up from 1 to 200 amu. Using RGA, this degassing procedure was determined to be sufficient to remove remaining solvents and water potentially obstructing N_2_ binding sites. N_2_ physisorption isotherms at 77 K performed in a Micromeritics ASAP 2020 were collected with 45 s equilibration time in the *p*/*p*_0_ range of 0–0.001 decreased to 10 s for *p*/*p*_0_ > 0.001. From these isotherms, the specific surface area was extracted through the Brunauer–Emmett–Teller (BET) model in the range of *p*/*p*_0_ from 10^–5^ to 0.1 and respecting the Rouquerol criterion.^50^ The pore size distribution and pore volume of the samples were modeled in the range of 5 × 10^–5^ < *p*/*p*_0_ < 0.8 using the commercially available DFT model for cylindrical geometries called “N_2_@77 K—oxide cylindrical pores Tarazona” available in the Micromeritics software.

### Optical Titration Measurements

For UV–vis titration measurements and photoluminescence (PL) quenching studies, C_60_⊂PCN-222­(H_2_,Zn) and PC_61_BM⊂PCN-222­(H_2_,Zn) suspension samples with a range of C_60_ or PC_61_BM concentration were prepared by adding 200 mL total volume aliquots of toluene containing 0.1–8 mol equiv of C_60_ or 0.1–16 mol equiv PC_61_BM per porphyrin linker to 10 mL PCN-222­(H_2_,Zn) suspensions in DMF. The final porphyrin linker concentration in each sample is 1.67 × 10^–6^ M. Samples were allowed to soak for 7 days to ensure diffusion of guest into pores prior to UV–vis and photoluminescence spectroscopy measurements.

### Transient Absorption Spectroscopy

MOF suspension samples were measured in a 2 mm optical path quartz cuvette. Transient absorption data sets were acquired using a Coherent Libra Ti:sapphire laser, with an output of 800 nm at 1 kHz. A Light Conversion TOPAS-C OPA was used to generate the ∼150 fs pump pulse tuned to 400 nm for these studies to excite near the peak of the porphyrin Soret band. The pump pulse energy was typically 100 nJ, and the pump spot size was found to be ∼300 μm through the use of a beam profiler (ThorLabs). In an Ultrafast Systems Helios Spectrometer, a small amount of 800 nm light was used to pump a 1 mm sapphire crystal to generate 450–800 nm probe light for UV–vis TA. NIR supercontinuum was generated in a 10-mm-thick sapphire crystal. A delay up to 5 ns can be achieved with the Helios. Two-dimensional (2D) maps were processed averaging three spectra for background and scattering light subtraction, chirp correction, and single-wavelength kinetic slices using Surface Xplorer. Data were then imported into MATLAB software to extract decay and species-associated spectra (SAS) using a custom global fitting routine.

## Computational Methods

Nudged elastic band (NEB)^51–53^ calculations are performed using the GFN2-xTB^54^ method in the ORCA^55,56^ program package. The previously reported C_60_ position^36^ centered within the triangularly arranged porphyrin linkers (P1) is taken as the start structure, and the position in between adjacent porphyrin trimers along the *c*-axis of the framework is taken as the final position (P2) of the NEB search. Due to the symmetry of the MOF along the *c*-axis, this search for the energetically lowest path from P1 to P2 covers all possible positions of the fullerene guest species along this pore. The calculation is performed with a cutout of the MOF as illustrated in Figures 4 and S9. The resulting energy curve (Figure S10) does not show any local minima between P1 and P2, allowing further investigations to focus on these two positions. Interaction energies between the C_60_ guest and the framework are calculated with PBEh-3c^57^ on GFN2-xTB geometries. For structures in which both P1 and P2 are occupied, interaction energies are calculated with and without a single explicit solvent molecule (DMF or 1,4-dioxane) located between the two C_60_ molecules, along with an implicit solvation model using SMD^58^ for the respective solvent. Molecular dynamics (MD) simulations with mcGFN-FF^59^ and NEB calculations with GFN2-xTB are performed to model the diffusion of these solvent or C_60_ guests from the small to large pores of the framework.

## Results and Discussion

### Synthesis and Characterization

PCN-222 nanoparticles were characterized by PXRD, SEM, and DLS (Figures S1 and S2). While diffraction measurements confirmed the crystallinity and phase of the framework, SEM revealed particle sizes of ∼120 nm. DLS characterization showed similar results, yielding an average particle size of ∼140 nm. The PCN-222 DMF suspensions display UV–vis absorption spectra typical of porphyrins like tetraphenylporphyrin (TPP) in solution (Figure S3). Notably, the Soret band for PCN-222 is red-shifted compared to that of analogous complex TPP in DMF. Postsynthetic metalation of the free base porphyrin linker sites of PCN-222 with Zn was first indicated by the distinct color change of the MOF nanoparticle suspension from magenta to violet and then confirmed by UV–vis absorption spectroscopy as revealed by the characteristic change in the Q-band region (Figure S3). Elemental analysis by AAS characterization is consistent with complete metalation of the porphyrin linkers (5.7 wt % Zn measured compared to the theoretical 5.2 wt %). Upon introduction of the fullerene guests (C_60_ or PC_61_BM), PXRD characterization confirmed the retention of crystallinity and phase of the PCN-222 hosts in each case (Figure S1).

### Characterization of Pore Volume Changes by N_2_ Physisorption

N_2_ physisorption measurements provide some insight into the location and distribution of the fullerene guest species in the MOFs. N_2_ physisorption isotherms and the results of pore size distribution analyses for PCN-222­(H_2_,Zn), C_60_⊂PCN-222­(H_2_,Zn), and PC_61_BM⊂PCN-222­(H_2_,Zn) are shown in Figure S4 and summarized in Table S1. Figure 1 summarizes the pore volume changes derived from these adsorption data when C_60_ is infiltrated in either the metal-free or Zn porphyrin versions of PCN-222. Similar results are found for PC_61_BM guests. The surface area and pore volume of the C_60_- and PC_61_BM-infiltrated MOFs are lower than those of the parent PCN-222 material, confirming the incorporation of these guest species within the porous frameworks. The pore size distribution analysis of these data reveals two different pore sizes consistent with the ∼1.2 nm-wide triangular porous channels and 3.7 nm-wide hexagonal porous channels of the PCN-222 framework. Notably, in the metal-free MOFs, the triangular pore volume decreased by nearly 59% while the hexagonal pore volume decreased by only 16% upon incorporation of C_60_. For PCN-222­(Zn), both the small and large pore volumes decreased by similar amounts, 24% for the triangular pores and 34% for the hexagonal pores, upon introduction of C_60_. The same volume reduction trends are observed for the PC_61_BM-loaded MOFs. The significantly larger reduction in small pore volume in PCN-222­(H_2_) upon incorporation of fullerene guests indicates higher loading of C_60_ or PC_61_BM in the triangular channels of this MOF compared to PCN-222­(Zn). This preferential adsorption may be a consequence of stronger binding affinity of the metal-free porphyrin linker sites for fullerene guests, as will be explored below.

**Figure 1:**
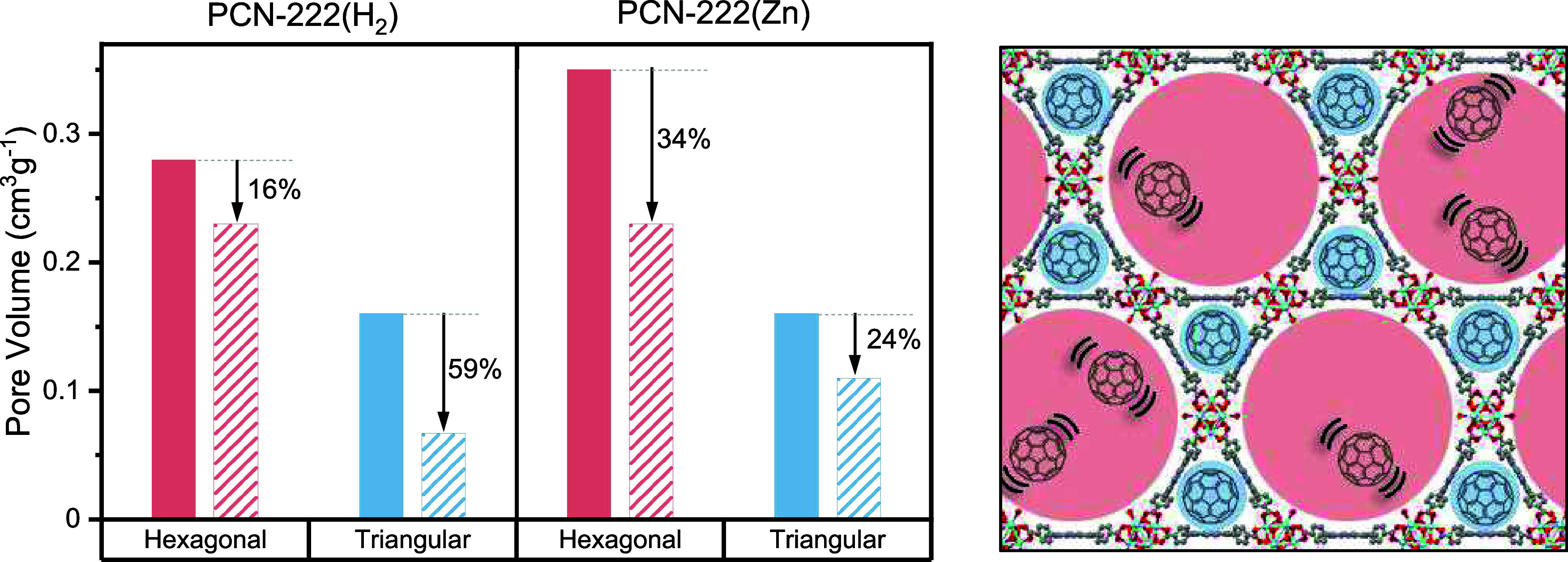
(Left) Pore volume of PCN-222­(H_2_,Zn) (solid) and C_60_⊂PCN-222­(H_2_,Zn) (patterned) in the hexagonal and triangular pores determined through fitting N_2_ physisorption isotherms. Arrow labels indicate the percent pore volume reduction upon introduction of C_60_ in PCN-222­(H_2_,Zn). (Right) Hexagonal (red) and triangular (blue) pores of the PCN-222 structure are illustrated, where the proposed location of C_60_ is graphically depicted and the weak interaction of C_60_ in the hexagonal pores is highlighted by using parentheses.

### Optical Electronic Spectroscopy Evidence of Fullerene–Porphyrin Linker Interaction

#### Photoluminescence Quenching

While the N_2_ physisorption characterization revealed the relative loading of the fullerene within the small triangular and large hexagonal pores of the framework, it does not offer direct evidence of interaction between the framework porphyrin linkers and fullerene guests. Photoluminescence quenching titration measurements provide initial confirmation of these binding interactions and insight into their relative strengths for the two MOFs and two fullerenes. Figure 2 illustrates the characteristic porphyrin linker emission of PCN-222­(H_2_) and PCN-222­(Zn), along with the quenching observed upon the introduction of C_60_ or PC_61_BM in each case. Compared to the analogous fluorescence titration measurements of TPP or ZnTPP in solution, which show negligible quenching upon fullerene titration (Figure S5), the fluorescence quenching behavior observed for the MOF systems indicates linker–guest interaction and implicates the unique confinement effect of the framework as its primary driving force. The PCN-222­(H_2_) framework exhibits more pronounced fluorescence quenching compared to PCN-222­(Zn) upon introduction of either C_60_ or PC_61_BM, indicating a greater association of the fullerene guests with the metal-free porphyrin linker sites compared with the Zn porphyrin analogues.

**Figure 2:**
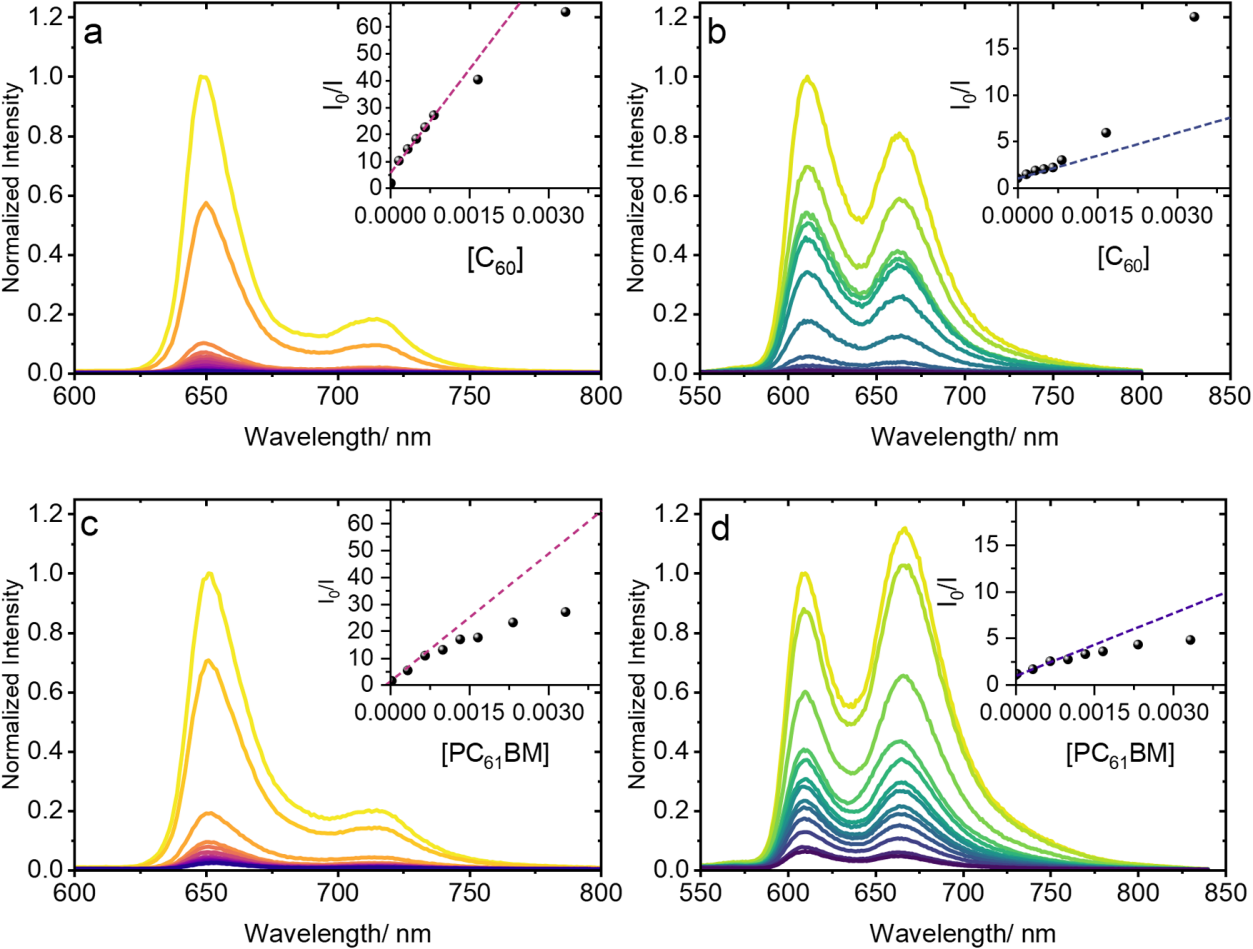
Fluorescence titration measurements of (a) C_60_⊂PCN-222­(H_2_), (b) C_60_⊂PCN-222­(Zn), (c) PC_61_BM⊂PCN-22_2_(H_2_), and (d) PC_61_BM⊂PCN-222­(Zn) in DMF. Insets: Stern–Volmer plots of *I*_0_/*I* as a function of the C_60_ or PC_61_BM concentration (mM).

The inset graphs of Figure 2 show Stern–Volmer plots derived from the titration measurements. The plots further illustrate the marked difference in fluorescence quenching between the metal-free and Zn porphyrin-based MOFs upon introduction of fullerene guests. Notable deviations from linearity are observed in the higher fullerene concentration range in each case. Interpreting these deviations, however, is complicated by the multiple factors likely influencing fluorescence quenching behavior in these materials. While combined static and dynamic quenching processes as well as sphere-of-action quenching are marked by upward curving Stern–Volmer plots, fractional accessibility of fluorophores yields the opposite trend, producing downward curvature of the plot with higher quencher concentration.^60^ In fluorescent MOFs such as PCN-222, all of these factors may be influencing the observed trends. Pore confinement, coupled with different fullerene–porphyrin ground state binding strengths that depend on the porphyrin metalation status, would likely influence the relative contribution of static, dynamic, and sphere-of-action fluorescence quenching by the fullerene guests, whereas incomplete diffusion of the fullerene guest molecules within the porous structure would render some framework porphyrin fluorophore linker sites effectively inaccessible to the fullerene guests. The net downward trend for the PCN-222­(H_2_) Stern–Volmer plot suggests that the latter factor dominates at higher C_60_ and PC_61_BM concentrations. However, the near-complete fluorescence quenching observed even at modest fullerene concentrations suggests that this population of inaccessible fluorophores is quite small. The overall upward curvature of the PCN-222­(Zn) Stern–Volmer plot at higher C_60_ concentration indicates the dominant effects of increased contribution of collision and/or sphere-of-action quenching between excited porphyrin linkers and weakly/nonbound, yet confined, fullerene guests. Evidence for this behavior can be found in a changing fluorescence lifetime with increased C_60_ loading (Figures S6 and S7). Interestingly, this upward curvature trend is not observed for PC_61_BM in PCN-222­(Zn). Its downward curving Stern–Volmer plot suggests that the added steric bulk of this fullerene guest may inhibit its diffusion, outweighing any collision or sphere-of-action quenching contributions.

#### UV–Vis Titration Measurements

UV–vis absorption spectroscopy characterization provides evidence for a ground state interaction between the fullerene guest and porphyrin linkers in PCN-222­(H_2_,Zn) through the observed red shift and absorbance decrease of the Soret band (Figure 3). These spectral changes occur when electron density is withdrawn from the porphyrin units by cofacial van der Waals interactions with the fullerene molecules within the porous framework.^61^ Note, no porphyrin-based Soret or Q-band spectral changes are observed upon addition of PC_61_BM or C_60_ to TPP or ZnTPP in DMF solutions (Figure S8), confirming the unique confinement effect of the porphyrin-based frameworks on the fullerene guests.

**Figure 3:**
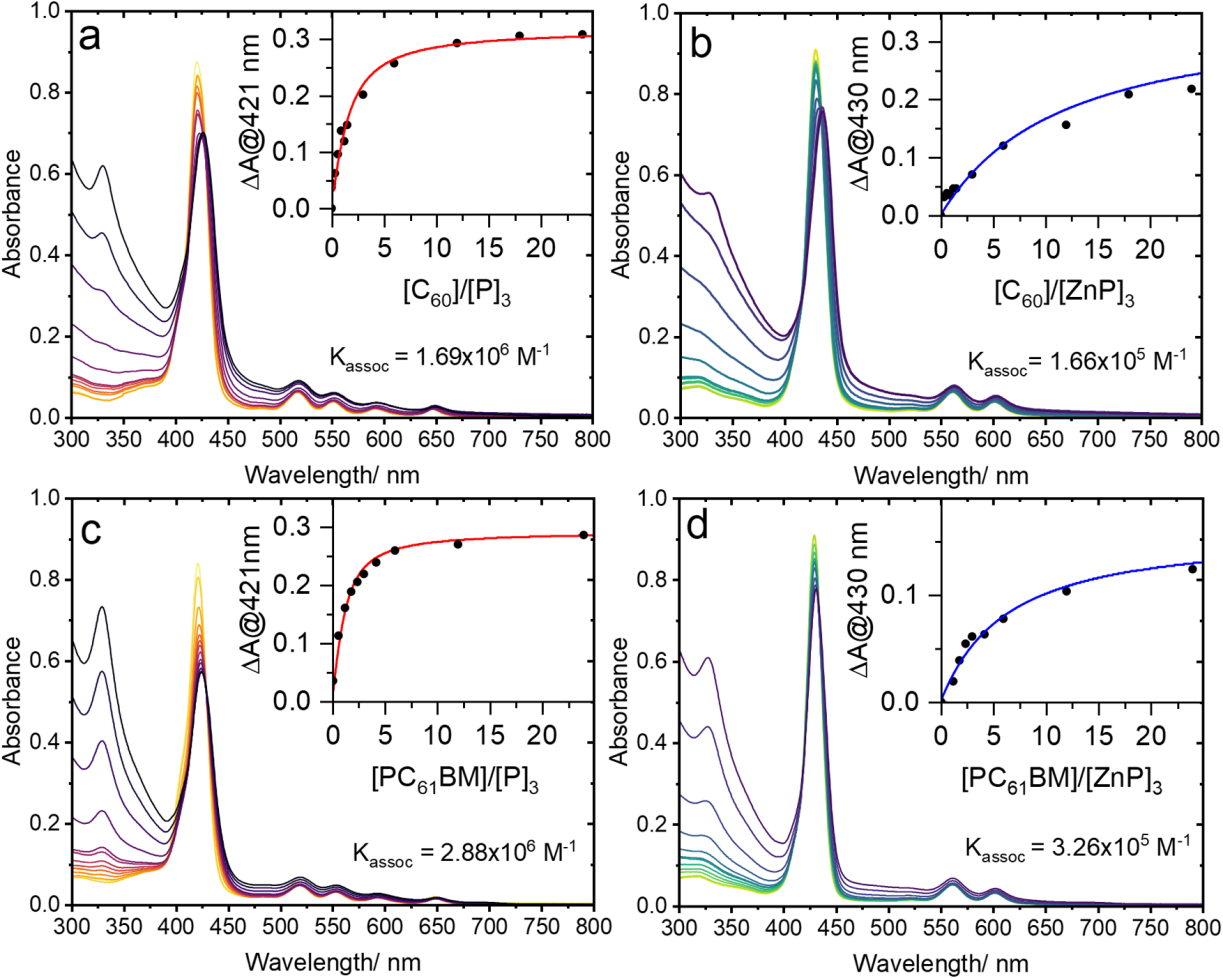
UV–vis titration measurements for (a) C_60_⊂PCN-222­(H_2_), (b) C_60_⊂PCN-222­(Zn), (c) PC_61_BM⊂PCN-222­(H_2_), and (d) PC_61_BM⊂PCN-222­(Zn) in DMF. Inset: binding isotherm generated by monitoring absorbance at λ = 421 nm (metal-free versions) and λ = 430 nm (Zn versions) as a function of relative fullerene/porphyrin concentration, expressed as fullerene to porphyrin trimer ratio, (e.g., [C_60_]/[P]_3_) in line with a triangular pore location.

UV–vis titration experiments (Figure 3) are used to establish association constants *K*_assoc_ for each fullerene-loaded MOF system. Titration isotherms are generated from the Soret band changes measured for the MOF suspensions upon introduction of the fullerene guests. On the basis of this analysis,^62,63^ the *K*_assoc_ values were evaluated to be 1.69 × 10^6^ M^–1^ for C_60_⊂PCN-222­(H_2_), 2.88 × 10^6^ M^–1^ for PC_61_BM⊂PCN-222­(H_2_), 1.66 × 10^5^ M^–1^ for C_60_⊂PCN-222­(Zn), and 3.26 × 10^5^ M^–1^ for PC_61_BM⊂PCN-222­(Zn). These results show that C_60_ and PC_61_BM have similar binding strengths within PCN-222. However, the metal-free porphyrin framework exhibits higher binding affinities for these fullerene species compared with the zinc analogue. While this trend contradicts the reported computational studies of C_60_ binding strength on this system,^36^ analogous titration studies of related metal-free and zinc porphyrin complexes with cofacial C_60_ interaction also showed stronger binding for the metal-free porphyrin versions.^64^ Notably, those studies similarly predicted a higher affinity for the Zn porphyrin version as well. The single inflection point in these titration data suggests that only one axial coordination site involves significant binding interaction. Based on the appropriately sized small triangular pores of the PCN-222 structure, we hypothesize that this is the location of the bound fullerene guest species. This binding location is both theoretically predicted^36^ and in line with reported cyclic porphyrin trimer supramolecular cage systems with similar dimensions that form analogous complexes with fullerenes.^65^ UV–vis titration measurements of that supramolecular system yielded association constants on the order of 10^4^–10^6^ M^–1^ depending on the solvent.

### Computational Investigation of Fullerene Locations and Solvation Environment

Computational modeling further elucidates the likely locations of the fullerene guest species within the triangular pore volume of the framework. In previous computational studies,^36^ only the position of the guest species that are cofacially associated with the porphyrin linkers in the small triangular pore has been considered. However, other positions along this channel might be occupied and could affect the properties of the MOF. To this extent, possible locations of the C_60_ molecule along the triangular porous channel of the PCN-222 structure were investigated by using the NEB reaction path exploration algorithm. Two minima were identified: one associated with the C_60_ interacting with the triangularly arranged porphyrin linkers (P1) and the other for the C_60_ located at the position between adjacent porphyrin sites along the triangular channel (P2) (see Figure 4).

**Figure 4:**
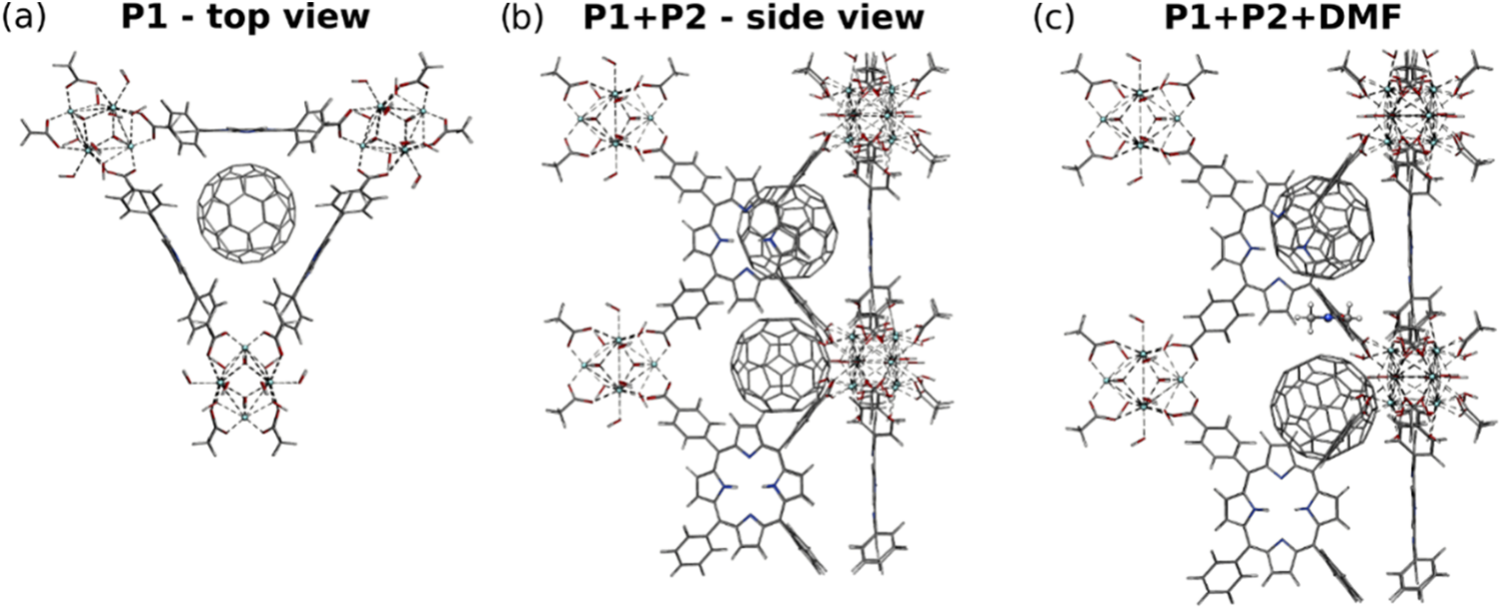
Illustration of C_60_ positions in the PCN-222 model system. (a) Top view along the triangular porous channel including a C_60_ molecule at position 1 in between the porphyrin linkers. (b) Side view of the model system with two C_60_ molecules located at positions 1 and 2. (c) Side view of the model system with a single DMF molecule between the two C_60_ at positions 1 and 2.

Calculated interaction energies, listed in Table 1, show that both positions can be occupied simultaneously without an energetic penalty. Specifically, the interaction energy with both positions occupied is 1.7–1.8 kcal/mol lower than the sum of the interaction energies of only P1 or P2 occupied depending on the implicit solvent. To investigate whether a solvation shell around the C_60_ molecules at P1 and P2 is feasible, we additionally calculated interaction energies with a single explicit solvent molecule between the two C_60_ molecules along with implicit solvation contributions. The energies reported in Table 1 show that with the addition of a DMF molecule, the interaction energy is reduced by 82%, and for 1,4-dioxane it is reduced by 108%. This indicates that occupying both positions in the small triangular pore with C_60_ molecules does not leave enough space for solvent molecules. Furthermore, molecular dynamics (MD) simulations showed that both solvent molecules can move from the small pore to the large pore through the gap between the two linkers in PCN-222, while the C_60_ molecules are unable to move through this gap. This suggests that upon fullerene loading, the small triangular pores are mostly occupied by the C_60_ molecules and the solvent molecules are displaced into the larger voids of the hexagonal pores. The results of the MD simulation are presented in Figures S11–S13 of the Supporting Information. The geometries for all calculations and the reaction equations for the interaction energies are given in the Supporting Information.

**Table 1:** Calculated Interaction Energies for C_60_ Molecules at the PBEh-3c Composite DFT Level on Optimized GFN2-xTB Geometries Located at Local Minima P1 and P2 within the One-Dimensional Triangular Pore of PCN-222 Model^a^

solvent	C_60_ location	energy (kcal/mol) (implicit solvent)	energy (kcal/mol) (implicit + explicit solvent)^b^
DMF	P1	–22.5	
	P2	–25.2	
	P1 + P2	–45.9	–8.5
1,4-dioxane	P1	–25.3	
	P2	–24.9	
	P1 + P2	–48.5	+3.7

a The energies include solvation contributions from the SMD model as indicated.

b A single solvent molecule located between the two C_60_ molecules as described in the text.

### Characterization of Charge Transfer and Recombination by Ultrafast Transient Absorption Spectroscopy

To analyze the excited state dynamics of these fullerene-loaded MOF systems, we turned to femtosecond transient absorption (TA) spectroscopy. TA data collected for C_60_⊂PCN-222­(H_2_) and C_60_⊂PCN-222­(Zn) are presented in Figure 5. The top panels (Figure 5a,b) contain spectral slices extracted at different time delays over the 5 ns window and kinetic traces (inset graphs) collected at diagnostic probe wavelengths associated with different transient species. The bottom panels (Figure 5c,d) show the species-associated spectra (SAS) of these two systems derived through the TA data global fitting analyses (vide infra). The TA results for the PC_61_BM-loaded MOFs are found in Figure S14, and those of the MOF nanoparticle suspensions without fullerene guests are shown in Figure S15. Upon photoexcitation, each MOF system displays characteristic porphyrin excited state absorption (ESA) at ∼480 nm along with Soret and Q-band ground state bleach (GSB) features, reflecting the immediate population of the S_1_ excited state of the porphyrin moiety (following fast internal conversion from S_2_ that occurs within the instrument response time). While PCN-222­(H_2_) and PCN-222­(Zn) in the absence of fullerene exhibit a decay of these features that indicates direct return to the ground state, the presence of the acceptor guest species in these MOFs yields TA spectral signatures that reflect CT excited state population along their decay path in each case. Following well-established literature precedent, the broad absorption band between 600 and 800 nm arises from the porphyrin radical cation,^42^ and the 1080 and 1020 nm ESA bands are due to the C_60_ and PC_61_BM radical anion^66^ species, respectively. By 5 ns, the broad visible ESA band converges into a feature centered around ∼740 nm, while the broad ESA in the NIR region beyond 900 nm decays to zero. This spectral evolution at longer delay times suggests charge recombination to the fullerene triplet excited state^67^ based on similar observations of electron transfer pathways in molecular porphyrin-C_60_ systems.^42^ At this long delay time, TA spectra for the metal-free porphyrin frameworks with fullerene guests also display residual absorption at ∼475 nm, accompanied by the GSB features, which is the hallmark of porphyrin locally excited triplet state population^68^ and has been documented in molecular porphyrin-C_60_ analogues.^37,42^ The persistence of both ESA features along with the GSB at long time delays in this case indicates that charge recombination occurs via both porphyrin and fullerene triplet excited state decay paths. In contrast, TA spectra for the Zn-metalated porphyrins in PCN-222­(Zn) with fullerene guests only show absorption around 740 nm by 5 ns, with all other features decaying to zero, indicating that the reverse charge transfer (RCT) event decays primarily to the C_60_ triplet state.

**Figure 5:**
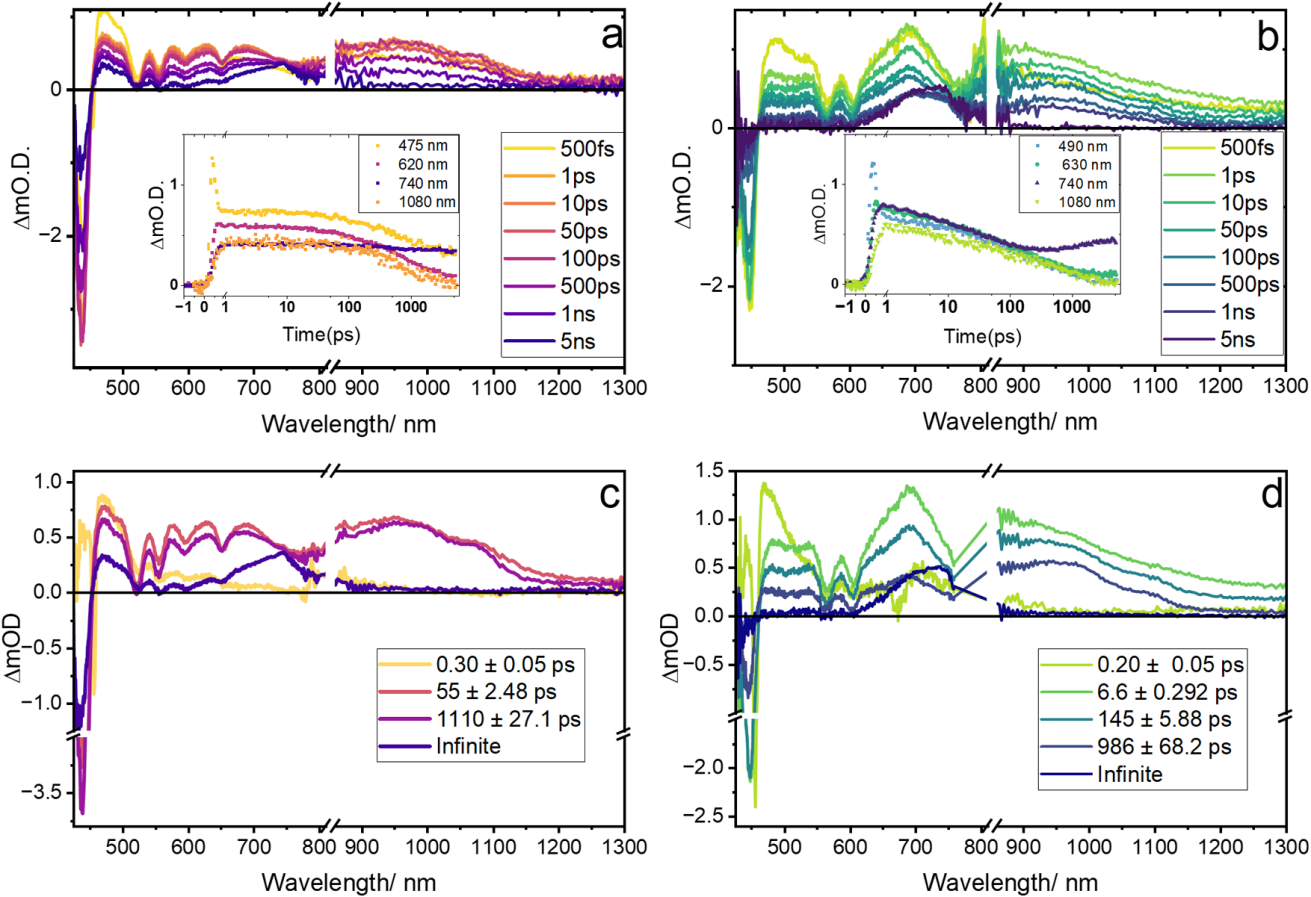
TA spectral overlays of (a) C_60_⊂PCN-222­(H_2_) and (b) C_60_⊂PCN-222­(Zn); the insets show kinetic slices obtained from the corresponding spectra at 475, 740, and 1080 nm. Species-associated spectra of (c) C_60_⊂PCN-22_2_(H_2_) and (d) C_60_⊂PCN-222­(Zn). Samples were measured as nanoparticle suspensions in DMF with λ = 400 nm excitation.

The global fits of the TA data provide further insight into the excited state decay pathways and kinetics for these fullerene guest-containing MOFs. Target analyses (see SI for more details) reveal four intermediate state components for both C_60_⊂PCN-222­(H_2_) and PC_61_BM⊂PCN-222­(H_2_) and five components for C_60_⊂PCN-222­(Zn) and PC_61_BM⊂PCN-222­(Zn). The SAS with the fastest lifetime component for both C_60_⊂PCN-222­(H_2_) and C_60_⊂PCN-222­(Zn) is assigned to the singlet excited state of the porphyrin donor linkers, ^1^P*-C_60_ or ^1^ZnP*-C_60_ with lifetimes τ = ∼0.3 and ∼0.2 ps, respectively. In C_60_⊂PCN-222­(H_2_), this initial porphyrin excited state decays to subsequent intermediate species, one with τ = ∼55 ps and another with τ = ∼1110 ps. These SAS components each display absorption features at ∼625 and ∼1080 nm (i.e., porphyrin cation and C_60_ anion spectroscopic signatures, respectively), indicating population of excited states with CT character. Notably, the τ = ∼55 ps SAS component displays broader spectral features that become slightly more resolved by the third component. These global fit results align with those reported for related porphyrin–fullerene molecular dyads that involve through-space electron transfer.^69–71^ In these systems, exciplex formation is invoked to explain the shorter lifetime component, indicating that its population precedes that of the formal charge transfer excited states along the decay pathway. The exciplex species in this molecular dyad, characterized by broader spectral features compared to the pure CT state, is a geometrical rearrangement of the donor and acceptor in the excited state that delocalizes the initial excitation of the donor with the possibility of some CT character. Thus, we assign the shorter and longer lifetime intermediate SAS components in C_60_⊂PCN-222­(H_2_) to the exciplex (PC_60_)* and P^+^C_60_^–^ CT state decay pathways, respectively. The C_60_⊂PCN-222­(Zn) excited state deactivation pathway also involves exciplex (ZnPC_60_)* and CT excited state, ZnP^+^C_60_^–^ populations; however, the exciplex involves two decay components (with τ_exciplex_ = 6.6 and 145 ps). These observed kinetics may be attributed to weaker fullerene binding affinities of the Zn porphyrin linkers (Figure 3), which allow multiple relaxation steps toward the optimal exciplex geometry. The formal CT excited state, with population similarly confirmed by the sharpened spectral features, has a lifetime of τ_RCT_ = 986 ps. The final “infinite” SAS component for C_60_⊂PCN-222­(H_2_) that persists beyond the 5 ns time window of the TA experiment, displays absorption features at ∼475 and ∼740 nm, corresponding to the triplet states of the porphyrin^68^ and C_60_,^67^ respectively. The final SAS component for C_60_⊂PCN-222­(Zn) only displays the ∼740 nm feature confirming exclusive decay via the C_60_ triplet excited state as previously discussed. A summary of the proposed excited state deactivation pathways derived from these TA analyses for the two MOF systems is depicted in Figure 6b.

**Figure 6:**
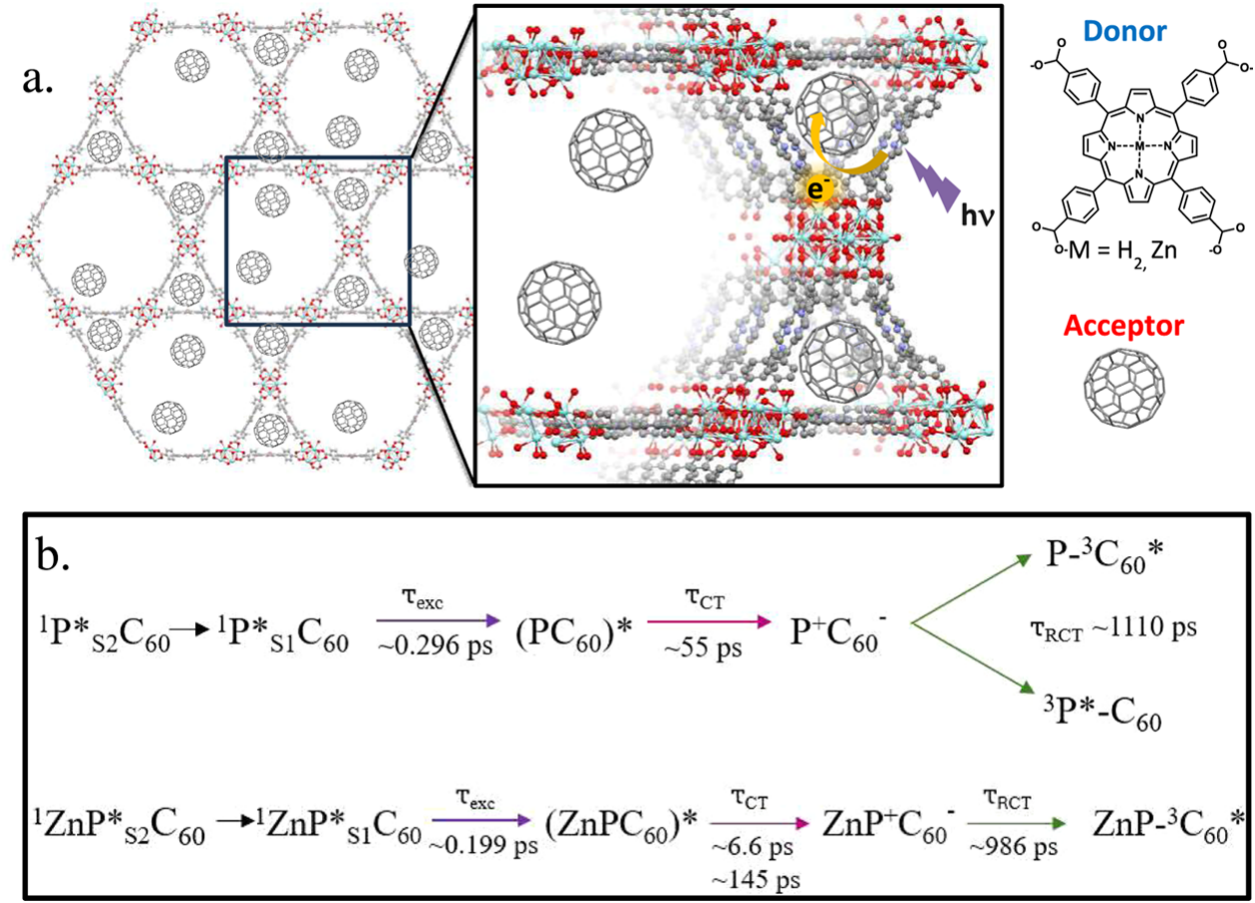
(a) Illustration of photoinduced electron transfer pathway as it relates to the proposed encapsulated location of the fullerene guests within the small triangular pores of the MOF. (b) Proposed excited state decay pathway of C_60_⊂PCN-222­(H_2_) (top) and C_60_⊂PCN-222­(Zn) (bottom).

Aside from the differences in exciplex and triplet decay pathways, both metal-free and zinc porphyrin frameworks loaded with C_60_ acceptor guests exhibit similar excited state dynamics with respect to charge transfer behavior and reverse charge transfer lifetimes (∼1 ns). The deviation in triplet decay pathways between C_60_⊂PCN-222­(H_2_) and C_60_⊂PCN-222­(Zn) can be rationalized based on the established triplet state energy levels of relevant porphyrin and zinc porphyrin complexes relative to that of C_60_. While the triplet energies reported for metal-free and zinc tetraphenylporphyrin complexes are 1.43 and 1.61 eV, respectively,^72^ that of the fullerene triplet state ^3^C_60_* is 1.55 eV.^73^ Assuming similar triplet state energies of these moieties in the MOF-C_60_ donor–acceptor systems, dual population of the lower-energy metal-free porphyrin triplet state along with ^3^C_60_* upon charge recombination is plausible, while the analogous population of the Zn porphyrin triplet state may be fleeting or energetically inaccessible. Furthermore, the significantly enhanced binding interaction and greater pore volume reduction for the fullerene-loaded PCN-222­(H_2_) framework compared to those of PCN-222­(Zn) are a potential source of divergent behavior. If the triplet energy migrates to more weakly bound fullerene in the large pore of PCN-222­(Zn), the proposed equilibrium between ^3^P* and ^3^C_60_* could be interrupted by subsequent fullerene diffusion that renders triplet energy transfer irreversible to ^3^P*. The global fit analyses of the PC_61_BM⊂PCN-222­(H_2_) and PC_61_BM⊂PCN-222­(Zn) TA results (Figures S19 and S20) reveal similar decay pathways and kinetics as the C_60_-loaded MOFs, including exciplex state formation, RCT lifetimes, and triplet state populations, indicating minimal impact of the butyric-methyl ester appendage on the electron transfer photophysics in this host–guest arrangement. This similar excited state behavior for the two fullerene intercalants, despite the apparent absence of a dynamic quenching channel for free or weakly bound PC_61_BM, suggests that energy offsets in the triplet manifolds may be the primary factor that dictates the product of reverse charge transfer.

The TA results presented thus far were obtained using DMF as the solvent environment for the fullerene-loaded framework suspensions. TA measurements were also collected for these MOF systems upon 1,4-dioxane solvent exchange to probe the influence of solvent polarity on their photophysical responses following the C_60_ introduction. Charge-separated excited state energies in most D–A systems are heavily influenced by solvent polarity, which can lead to drastic changes in observed photoinduced electron transfer and recombination pathways.^44,69,74–79^ While polar or moderately polar solvents such as DMF can facilitate charge separation by stabilizing the CT state, nonpolar media can hinder photoinduced charge separation by rendering the CT state less energetically favorable, or even inaccessible. The TA data collected in 1,4-dioxane are shown in Figure S16 with the results of the global fitting analysis depicted in Figures S21 and S22. Using the same sequence models applied to these systems measured in DMF, global fitting yielded analogous intermediate SAS components associated with singlet porphyrin-localized excited state, exciplex, and CT excited state populations. Furthermore, the TA analyses reveal comparable charge recombination pathways dominated by C_60_ triplet excited state population for C_60_⊂PCN-222­(Zn) or a dual population of both porphyrin and C_60_ triplet excited states for C_60_⊂PCN-222­(H_2_). Notably, despite the significant difference in solvent polarity between DMF (ε = 36.7) and 1,4-dioxane (ε = 2.20) environments, the TA results revealed similar CT state formation and decay lifetimes for these fullerene-loaded MOF materials in both solvents. This suggests that the C_60_ acceptor species are sufficiently shielded when confined within the small triangular channels of the framework, such that the resulting D–A CT excited state energies are essentially impervious to the surrounding solvent environment. As a result, only the electrostatic interaction between the framework porphyrin linkers and fullerene guest species influences their electron transfer behavior and kinetics. The expulsion of solvent from the small triangular channels upon introduction of the fullerene guests is further supported by the computational results presented above, which reveal that the C_60_-loaded MOF in the absence of solvent is more thermodynamically favorable than this system in the presence of interstitial DMF or 1,4-dioxane solvent molecules.

### Comparison with Molecular Porphyrin–Fullerene D–A Systems

Comparing electronic structure and excited state dynamics derived from the observed photophysics of these fullerene-loaded MOF materials with those of relevant molecular porphyrin–fullerene D–A systems highlights the unique role of the framework in dictating photoinduced electron transfer and decay pathways. It is well established that covalently linked C_60_-porphyrin­(Zn,H_2_) dyads undergo D–A electron transfer following local porphyrin donor excitation, with charge separation and recombination pathways and kinetics determined by the linker distance between the porphyrin donor and fullerene acceptor moieties as well as the solvation environment.^44,69,74,75^ In these molecular examples, however, the fullerene and porphyrin moieties are connected via a single linker, which leads to substantial conformational flexibility and negligible direct π–π interaction between the porphyrin plane and the convex π surface of the fullerene. Cyclophane-like C_60_-porphyrin dyads, with two separate linkers symmetrically connecting the porphyrin and fullerene components, yield face-to-face π-stacked structures,^61,70^ while cage complexes such as the covalent organic polyhedron, COP-5, possess a fixed cofacial orientation of two porphyrin sites for encapsulation of fullerene (C_70_ in the reported system).^37^ These molecular analogues display through-space π–π interactions that in some ways resemble those of the fullerene-loaded porphyrin-based MOFs. In each molecular case, direct interchromophore interaction, with similar fixed edge-to-edge donor–acceptor distances, *R*_EE_ ∼2.8 Å, is evinced by absorption spectra perturbations like those observed in C_60_⊂ and PC_61_BM⊂PCN-222­(H_2_,Zn). Unlike the MOF systems, however, the molecular analogues exhibit additional NIR absorption and emission features (at least in nonpolar solvents). The appearance of the new absorption band indicates a direct excitation to the CT excited state, while the emission band is attributed to its radiative decay.^37,61^ For the cyclophane-like dyads, despite the lack of this CT emission in polar solvents, exciplex formation is assigned as a precursor to the fully charge-separated state, as mentioned above. In the C_70_@COP-5 system, however, the charge transfer state forms from the COP-5 porphyrin singlet excited state without evidence of an intervening exciplex, regardless of solvent polarity. While the PCN-222 framework rigidity and cage-like encapsulation of the fullerene guest molecules might predict similar behavior, exciplex intermediate is nonetheless detected in this system in both polar and nonpolar solvent environments. The different pathways may be attributed to the fullerene triangular pore location and subsequent electronic coupling with three porphyrin moieties in PCN-222 (rather than two in the case of COP-5) as well as the different fullerene species encapsulated in each case (C_60_ vs C_70_). Regardless of the participation of exciplex states, the electron transfer process occurs on the ultrafast time scale in each case.

The reverse charge transfer lifetime trends observed for the C_60_⊂ and PC_61_BM⊂PCN-222­(H_2_,Zn) series resemble the behavior reported for the fullerene-loaded COP-5 system. Namely, electron transfer occurs for the cage complex in both polar and nonpolar solvents with similar charge recombination lifetimes, despite a large difference in solvent dielectric constant. The prolonged CR lifetimes are attributed to the rigidity of the porphyrin cage in COP-5 and very small reorganization energy upon photoinduced electron transfer. Framework rigidity in the PCN-222 series likely plays a similar role along with the solvent-excluded pore location of the acceptor species, which essentially eliminates the solvent reorganization effects on the energetics of the CT state and, therefore, the rate of charge recombination. Notably, the lifetime is even longer than the 300–600 ps range reported for the COP-5 system, and more impervious to solvent polarity with τ_RCT_ ∼1 ns for all MOF systems in both polar and nonpolar solvents. The deviation in charge recombination pathways further highlights how the unique arrangement, rigidity, and solvent exposure of the fullerene and porphyrin moieties in each case influence the relative energy of the CT excited state. The cofacial C_60_-porphyrin molecular systems that form charge-separated excited states in polar solvents decay via direct recombination to the ground state, bypassing any triplet excited state population. Only in the C_70_@COP-5 system with a nonpolar solvent environment is a porphyrin triplet state invoked as the dominant charge recombination pathway. The dual triplet decay pathway observed upon charge recombination within the fullerene-loaded PCN-222­(H_2_) framework suggests near-energetic resonance between the CT state and both porphyrin and fullerene triplets.

## Conclusions

The framework-imposed arrangement of porphyrin linkers in PCN-222 is responsible for the unique donor–acceptor interactions with encapsulated fullerene guests. The distinct occupation, binding strength, and solvent accessibility of acceptors in different pore types of these tailored MOFs lead to electronic structure perturbations that are unmatched in molecular analogues. Although in all cases fast charge transfer occurs from porphyrin to fullerene, the subsequent flow of energy and charge is redirected by the tunable energy landscape—most notably, through varying excimer relaxation pathways and an eventual exclusive formation of fullerene triplet in the Zn-metalated MOF. Employing this energy flow strategically in a photochemical or catalytic reaction is a future goal that may require additional structural optimization and the inclusion of catalytic metal species.

## Supplementary Material





## Notes

Powder XRD, SEM, DLS, UV–vis and fluorescence titration control experiments, N_2_ physisorption isotherms, pore size distributions, further computational results, additional TA results, details of global fitting procedure, and global fitting analysis results (PDF)
Geometries for interaction energies, MD trajectories, and NEB trajectories (ZIP)
