# Supplementary material for: Charge Transfer and Recombination Pathways through Fullerene Guests in Porphyrin-Based MOFs
Source: J Phys Chem C Nanomater Interfaces. 2025 Apr 23;129(17):8215–27. doi: 10.1021/acs.jpcc.5c00161 (PMC12333347; doi:10.1021/acs.jpcc.5c00161)
Supplement: Supplementary file 1 [file jp5c00161_si_001.pdf]

Charge Transfer and Recombination Pathways through Fullerene Guests in Porphyrin-based MOFs

Alison Arissa[1], Thomas Rose[2], Noémi Leick[3], Stefan Grimme[2], Justin C. Johnson*[3], Jenny V. Lockard*[1]

[1]Department of Chemistry, Rutgers University-Newark, Newark, New Jersey 07102, USA

[2]Mulliken Center for Theoretical Chemistry, Clausius-Institut für Physikalische und Theoretische Chemie, Rheinische Friedrich-Wilhelms Universität Bonn, Bonn 53115, Germany

[3]National Renewable Energy Laboratory, 15013 Denver West Parkway, Golden, Colorado 80401, USA

Supporting Information

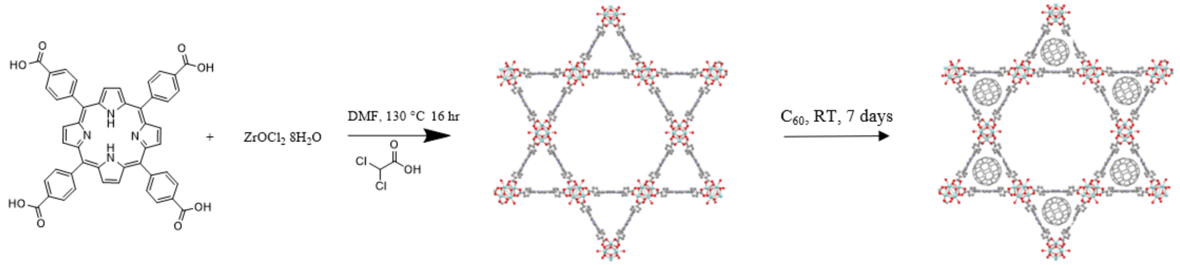

Scheme S1 PCN-222 synthesis and post-synthetic modification with C_{60}.

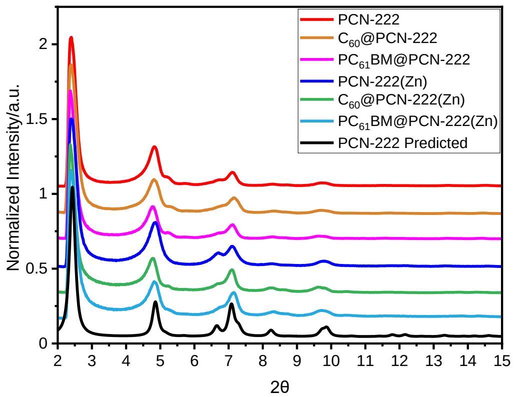

Figure S1: Powder XRD patterns of PCN-222 (red), C_{60}⊂PCN-222 (orange), $PC_{61}BM$⊂PCN-222 (pink), PCN-222(Zn) (blue), C_{60}⊂PCN-222(Zn) (green), $PC_{61}BM$⊂PCN-222(Zn) (green), along with the theoretical pattern derived from the reported crystal structure for PCN-222[1](black).

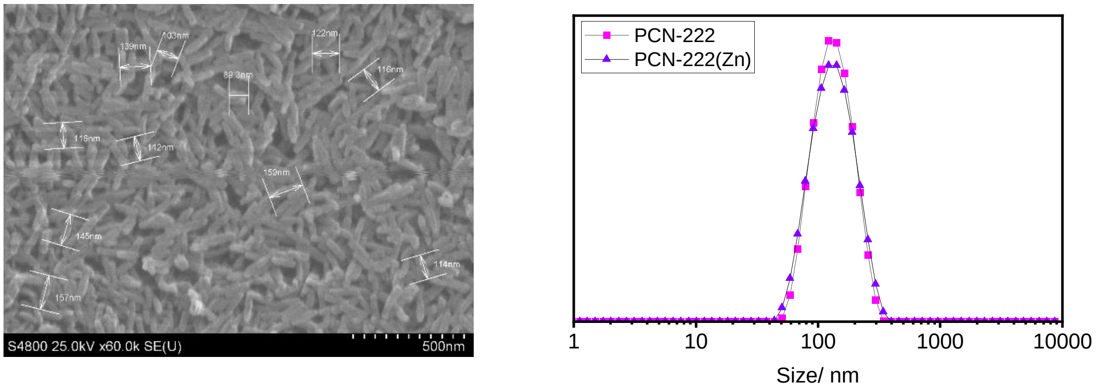

Figure S2: SEM of PCN-222(H_2) (left), DLS of PCN-222(H_2) and PCN-222(Zn) (right).

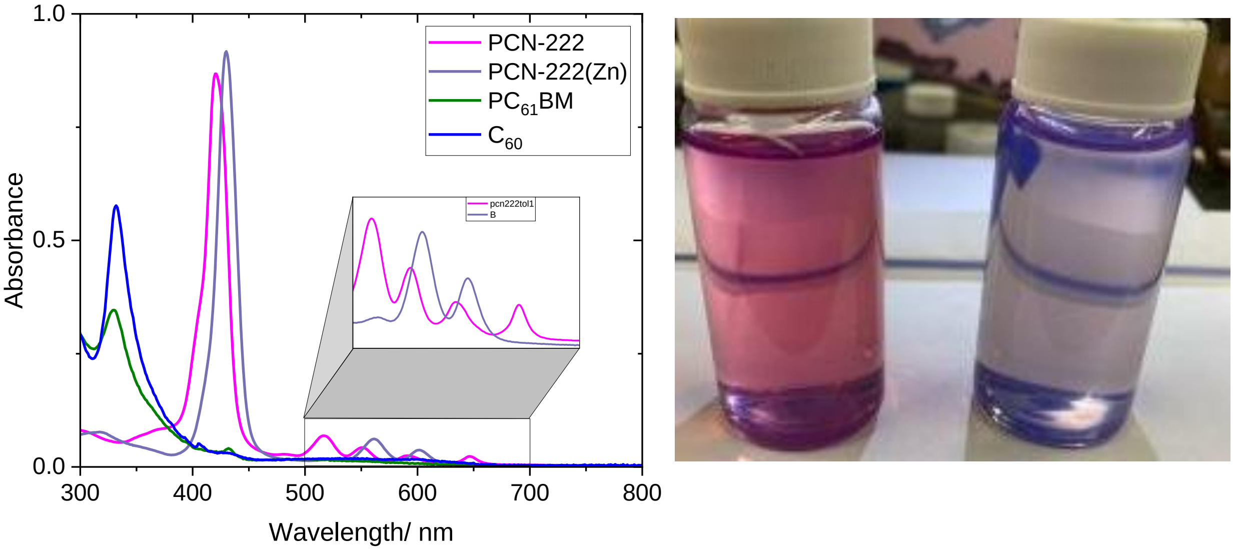

Figure S3: a) UV-Vis spectra of $PC_{61}BM$, C_{60}, PCN-222(H_2) and PCN-222(Zn) nanoparticle suspensions. Inset: magnification of Q-band region b) photograph illustrating MOF nanoparticle suspensions and color change from magenta to violet upon Zn metalation of PCN-222(H_2).

Nitrogen physisorption results:

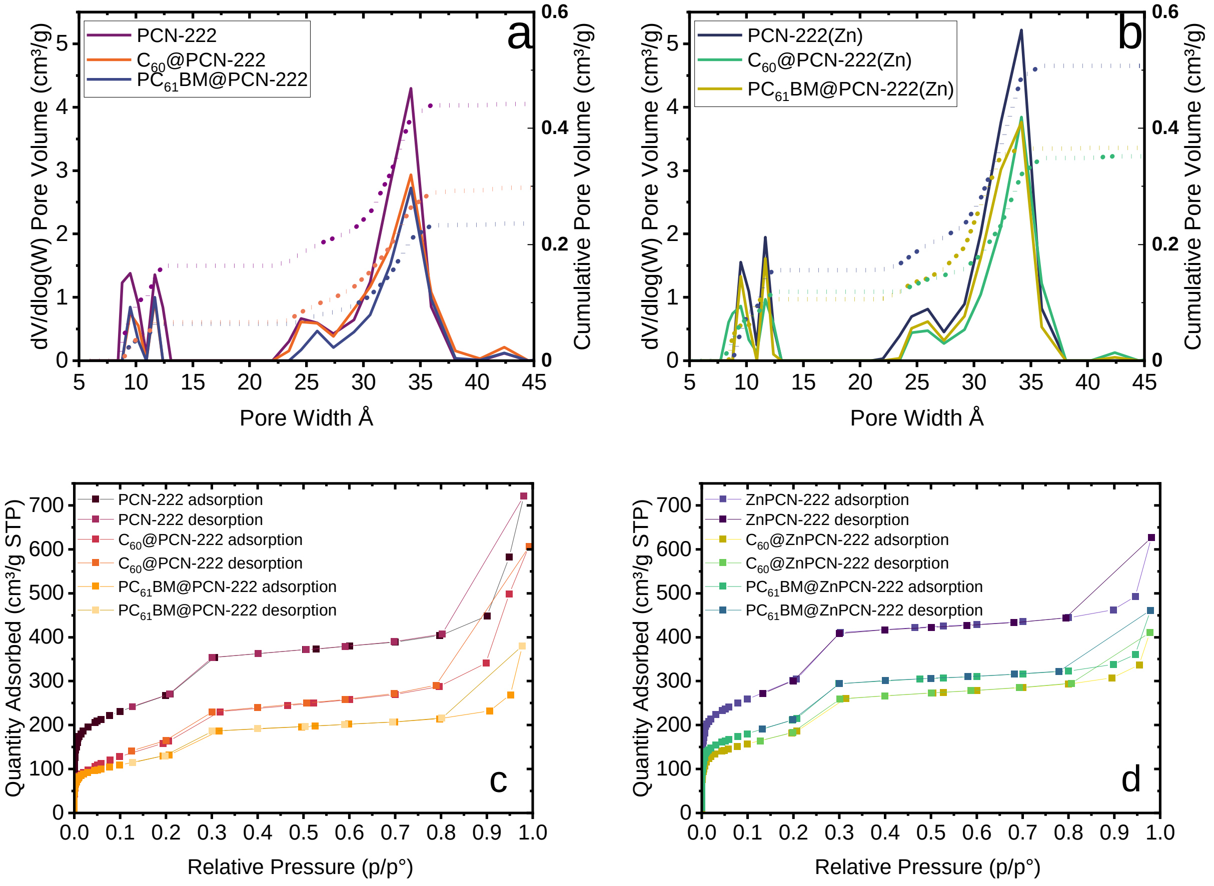

Figure S4 Pore size distribution of a) PCN-222(H$_2$), C$_{60}$⊂PCN-222(H$_2$), PC$_{61}$BM⊂PCN-222(H$_2$) b) PCN-222(Zn), C$_{60}$⊂PCN-222(Zn), PC$_{61}$BM⊂PCN-222(Zn) and N$_2$ isotherms of c) PCN-222(H$_2$), C$_{60}$⊂PCN-222(H$_2$), PC$_{61}$BM⊂PCN-222(H$_2$) d) PCN-222(Zn), C$_{60}$⊂PCN-222(Zn), PC$_{61}$BM⊂PCN-222(Zn)

Table S1 Triangular and hexagonal pore volumes and total surface areas of PCN-222(H$_2$,Zn) before and after fullerene introduction derived from N$_2$ physisorption measurements.

	Triangular cm^{3}g^{-1}	Hexagonal cm^{3}g^{-1}	Surface Area m^2/g
PCN-222(H$_2$)	0.16	0.28	863.5 ± 5.1
C$_{60}$⊂PCN-222(H$_2$)	0.067	0.23	482.0 ± 7.1
PC$_{61}$BM⊂PCN-222(H$_2$)	0.064	0.17	442.3 ± 4.3
PCN-222(Zn)	0.16	0.35	939.5 ± 12.2
C$_{60}$⊂PCN-222(Zn)	0.12	0.23	630.1 ± 3.9
PC$_{61}$BM⊂PCN-222(Zn)	0.11	0.26	668.1 ± 7.9

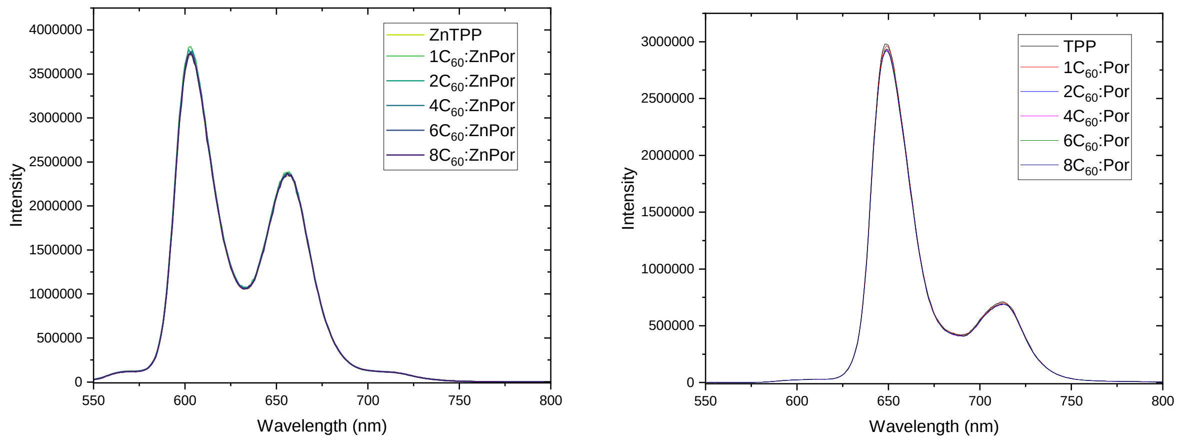

Figure S5 Fluorescence titration of a) TPP and b) ZnTPP in DMF with aliquots of C_{60} toluene solution

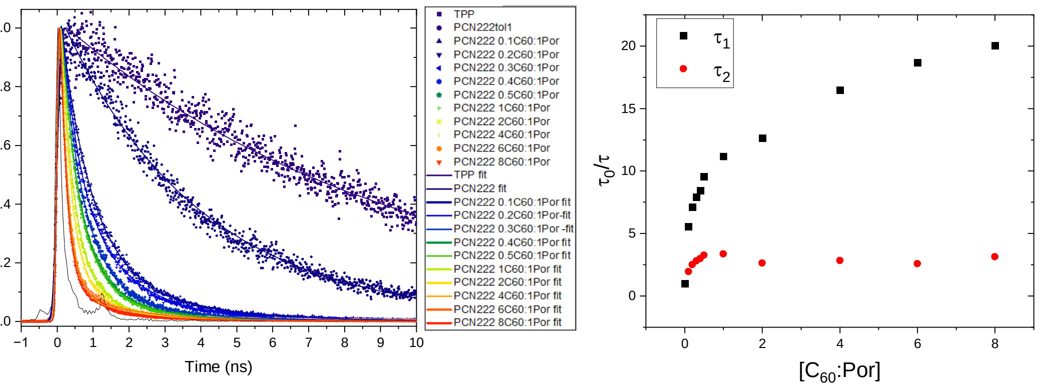

Figure S6 Left: Time correlated single photon counting measurements of C_{60}⊂PCN-222(H_2) Right: Stern-Volmer plots of t_0/t as a function of C_{60} concentration (C_{60}:Por ratio). Solvent: DMF

Table S2. Summary of Fluorescence lifetime data for C_{60}⊂PCN-222(H_2) system

**Sample**	**τ_1 (ns)**	**A_1**	**τ_2 (ns)**	**A_2**
**TPP**	9.56 ± 0.04	1.03 ± 0.002		
**PCN-222**	3.82 ± 0.01	1.06 ± 0.002		
**0.1C60:Por**	0.689 ± 0.008	0.749± 0.008	1.97 ± 0.02	0.423 ± 0.009
**0.2C60:Por**	0.537 ± 0.005	0.817± 0.008	1.52 ± 0.02	0.399 ± 0.009
**0.3C60:Por**	0.482 ± 0.005	0.839 ± 0.008	1.36 ± 0.01	0.401 ± 0.009
**0.4C60:Por**	0.453 ± 0.003	0.963 ± 0.006	1.28 ± 0.01	0.288 ± 0.007
**0.5C60:Por**	0.399 ± 0.003	0.932± 0.006	1.17 ± 0.01	0.369 ± 0.007
**1C60:Por**	0.342 ± 0.002	1.10± 0.004	1.13 ± 0.01	0.238 ± 0.005
**2C60:Por**	0.302 ± 0.001	1.21± 0.002	1.46 ± 0.02	0.151 ± 0.002
**4C60:Por**	0.232± 0.001	1.32 ± 0.002	1.35 ± 0.01	0.141 ± 0.002
**6C60:Por**	0.204 ± 0.001	1.37± 0.003	1.48 ± 0.01	0.144 ± 0.001
**8C60:Por**	0.190± 0.001	1.41 ± 0.002	1.22 ± 0.01	0.143 ± 0.001

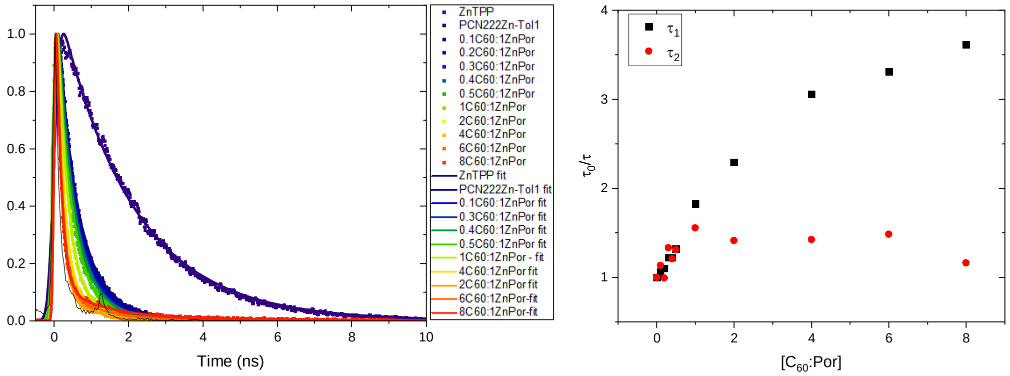

Figure S7 Left: Time correlated single photon counting measurement of C_{60}⊂PCN-222(Zn) Right: Stern-Volmer plots of t_0/t as a function of C_{60} concentration (C_{60}:Por ratio). Solvent: DMF

Table S3. Summary of Fluorescence lifetime data for C_{60}⊂PCN-222(Zn) system

**Sample**	**τ_1**	**A_1**	**τ_2**	**A_2**
**ZnTPP**	1.993 ± 0.003	1.16 ± 0.001		
**PCN-222(Zn)**	0.512 ± 0.002	1.18 ± 0.004	1.49 ± 0.04	0.093 ± 0.005
**0.1C60:Por**	0.483 ± 0.003	1.19 ± 0.01	1.32 ± 0.05	0.107 ± 0.009
**0.2C60:Por**	0.465 ± 0.002	1.23 ± 0.004	1.50 ± 0.06	0.068 ± 0.005
**0.3C60:Por**	0.418 ± 0.002	1.19 ± 0.01	1.12 ± 0.03	0.138 ± 0.008
**0.4C60:Por**	0.418 ± 0.002	1.21 ± 0.01	1.23 ± 0.04	0.106 ± 0.007
**0.5C60:Por**	0.388 ± 0.002	1.16 ± 0.01	1.14 ± 0.03	0.118 ± 0.007
**1C60:Por**	0.280 ± 0.001	1.33 ± 0.004	0.959 ± 0.020	0.116 ± 0.005
**2C60:Por**	0.224 ± 0.001	1.30 ± 0.004	1.05 ± 0.03	0.087 ± 0.004
**4C60:Por**	0.167 ± 0.001	1.43 ± 0.003	1.05 ± 0.03	0.072 ± 0.002
**6C60:Por**	0.155 ± 0.001	1.43 ± 0.002	1.01 ± 0.01	0.127 ± 0.002
**8C60:Por**	0.142 ± 0.001	1.42 ± 0.003	1.28 ± 0.01	0.126 ± 0.001

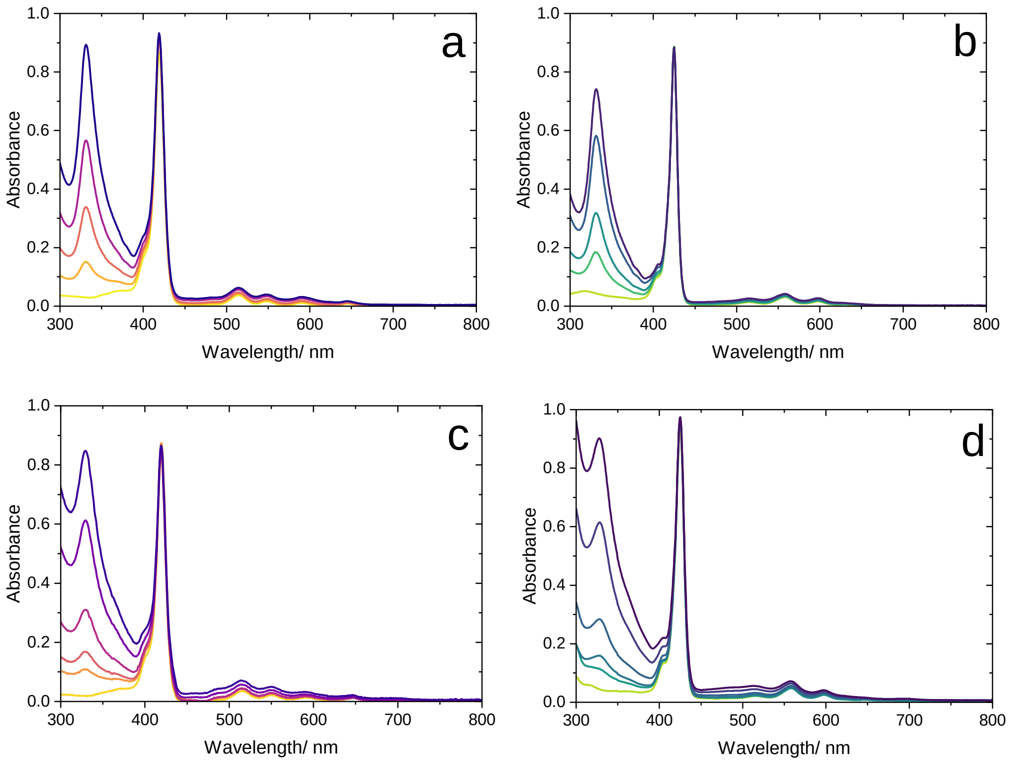

Figure S8. UV-Vis titration of control experiments in which toluene aliquots of C_{60} were added to DMF solutions of a) TPP and b) ZnTPP and aliquots of $PC_{61}BM$ were added to DMF solutions c) TPP and d) ZnTPP.

<u>Computational Results:</u>

Reaction equations for the interaction energies listed in Table 1 of the manuscript.
The interaction energy with only the implicit solvent is calculated according to:

$$C_{60} + MOF_{PCN222} \rightarrow C_{60}@MOF_{PCN222}$$

The interaction energy with additional explicit solvent is calculated according to:

$$2C_{60} + MOF_{PCN222} + DMF \rightarrow (2C_{60}, DMF)@MOF_{PCN222}$$

or

$$2C_{60} + MOF_{PCN222} + dioxane \rightarrow (2C_{60}, dioxane)@MOF_{PCN222}$$

All PBEh-3c energy calculations include solvation contributions from the implicit solvation model SMD for the respective solvent. The MD simulations with mcGFN-FF use a modified version with increased Zr-bond strength parameter to prevent the dissociation of an OH group from a Zr-cluster. This modification does not influence the non-covalent interactions.

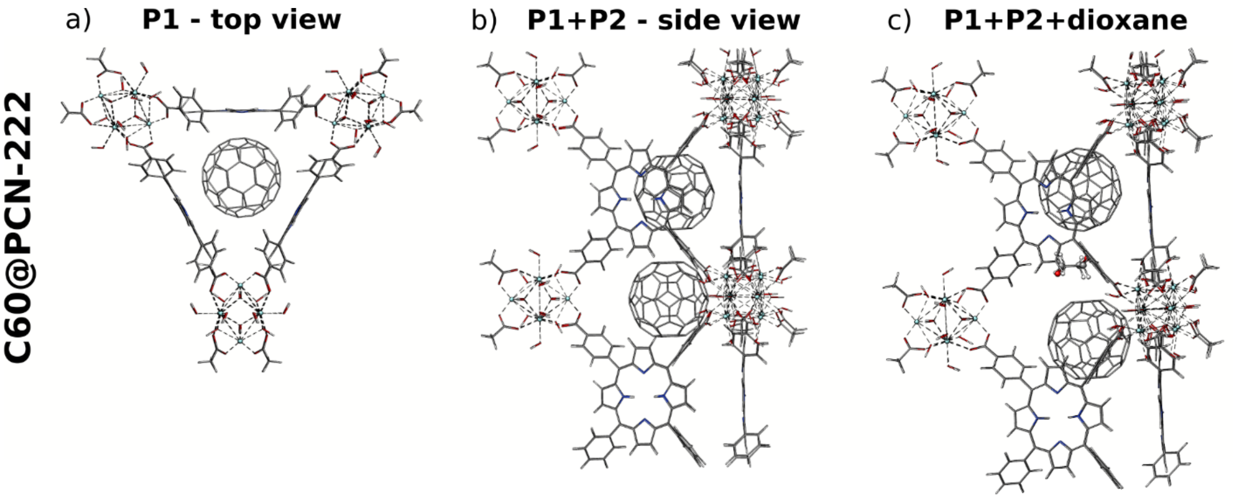

Figure S9 Illustration of C_{60} positions in the PCN-222 model system. a) Top view along the triangular porous channel including a C_{60} molecule at position 1 in between the porphyrin linkers. b) Side view of the model system with two C_{60} molecules located at positions 1 and 2. c) Side view of the model system with a single 1,4-dioxane molecule between the two C_{60} at positions 1 and 2.

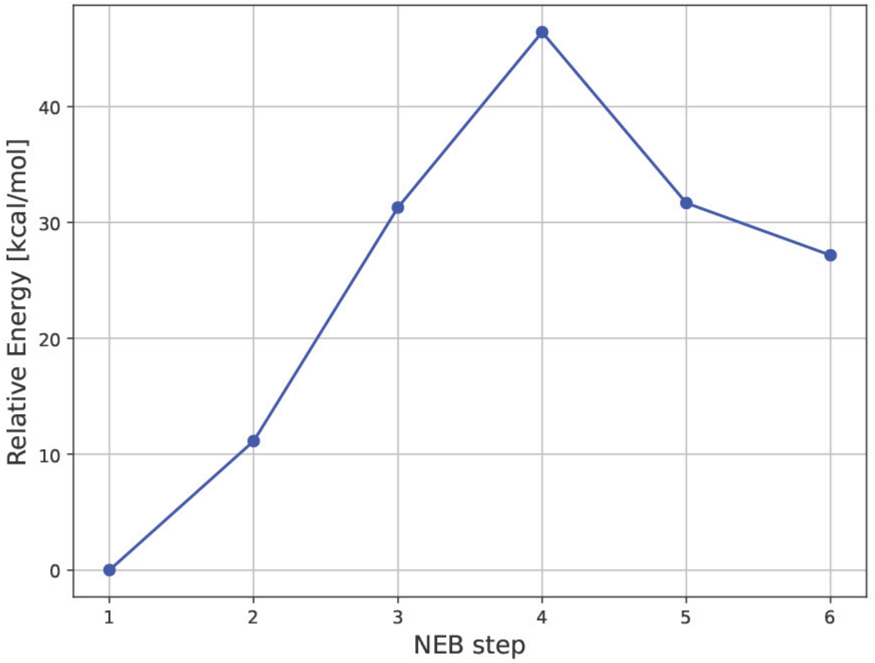

Figure S10 Energies of a GFN2-xTB NEB calculation relative to the energy for position 1. Note that the goal of this calculation is to search for further minima between positions 1 and 2. The difference between the relative energy at position 1 (NEB step 1) and position 2 (NEB step 6) is approximately 20 kcal/mol larger than the difference of the hybrid DFT values in Table 1. Therefore, the barrier is likely overestimated by a similar amount. However, the progression does not indicate another minima and due to the symmetry of the MOF along the c-axis, there are now further positions in the small triangular channel that should be investigated.

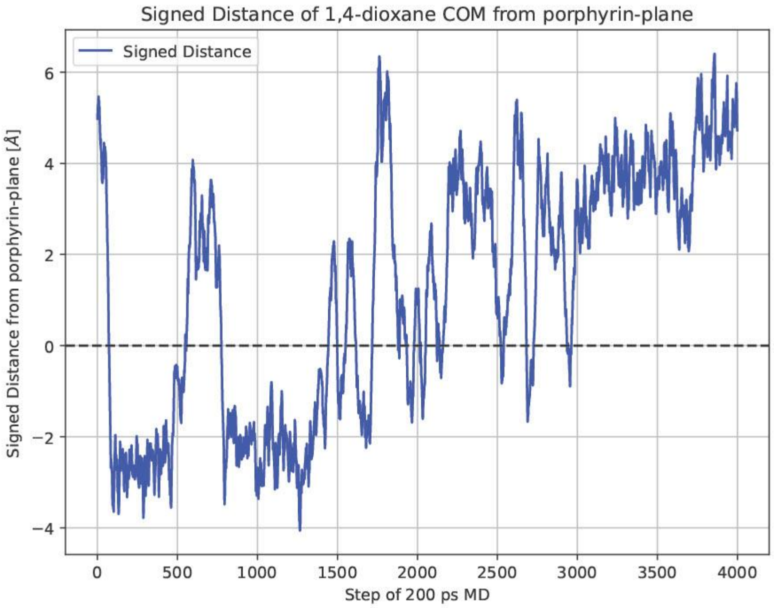

Figure S11 Distance of the COM of 1,4-dioxane from one of the porphyrin planes in the PCN-222 framework during mcGFN-FF MD simulation. A negative sign means that the COM is in the large pore and a positive sign corresponds to the small triangular pore.

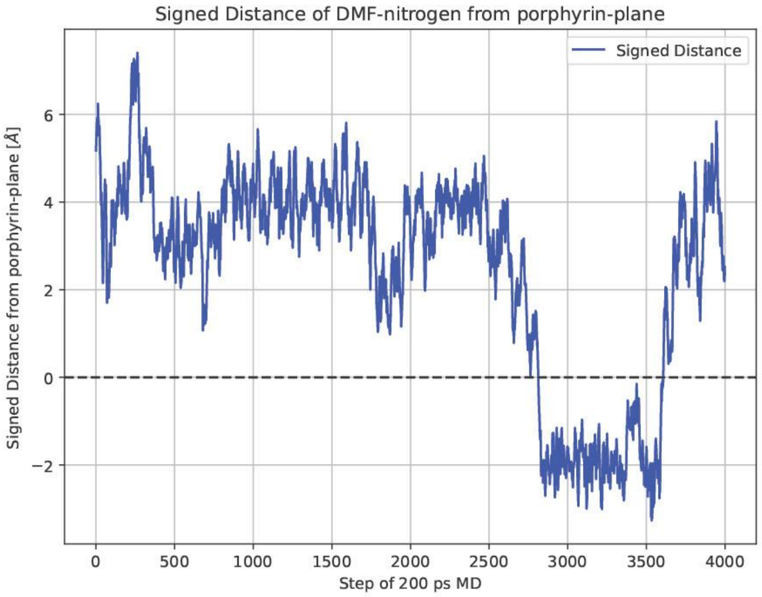

Figure S12 Distance of the nitrogen in DMF from one of the porphyrin planes in the PCN-222 framework during mcGFN-FF MD simulation. A negative sign means that the COM is in the large pore and a positive sign corresponds to the small triangular pore.

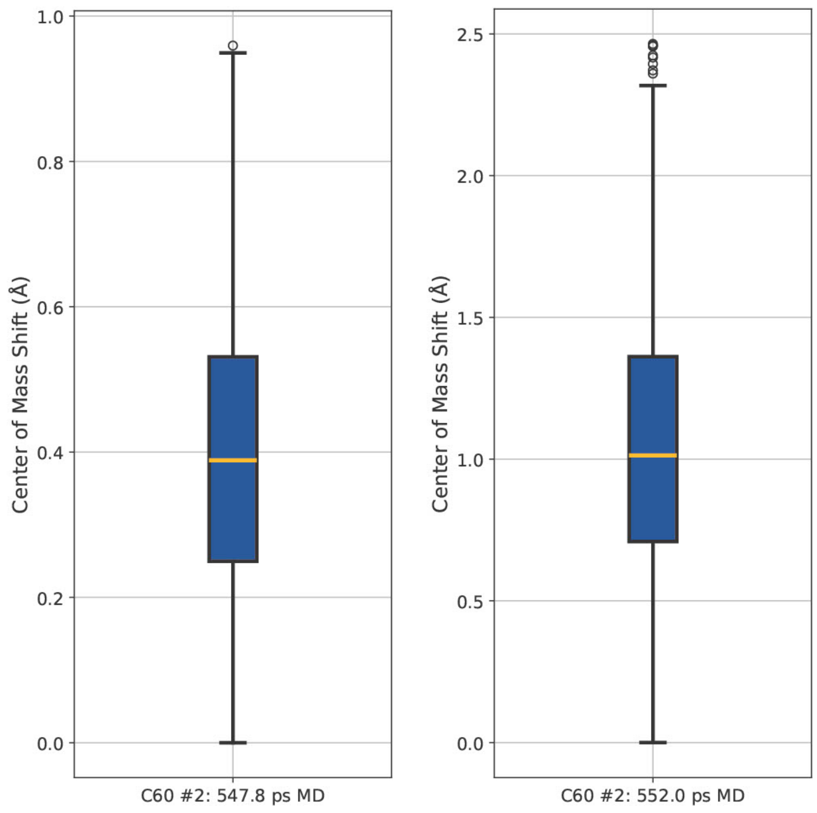

Figure S13 Boxplot for distances from the initial Center of mass (COM) of the C_{60} molecule for all geometries from a mcGFN-FF MD simulation. The MD on the left side starts at position 1 and the MD on the left side starts at position 2. While the C_{60} molecule can move around more in position 2, it does not move to the other position for either starting point. The simulations are approximately 550 ps long as indicated in the subfigures.

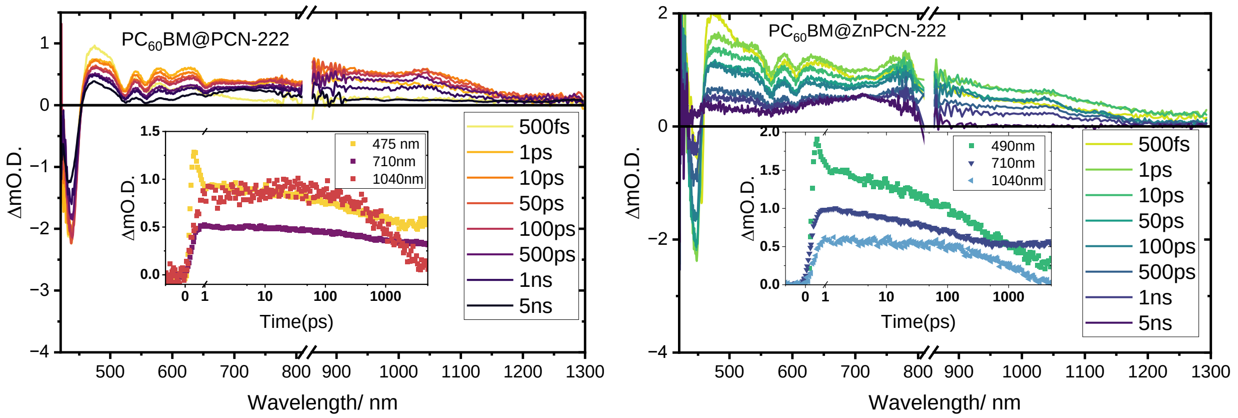

Figure S14 TA spectral overlays of a) $PC_{61}BM\subset PCN$-222(H_2) and b) $PC_{61}BM\subset PCN$-222(Zn), insets show kinetic slices obtained from the corresponding spectra at 475, 740 and 1080 nm for $PC_{61}BM\subset PCN$-222(H_2) and 490, 740 and 1080 nm for $PC_{61}BM\subset PCN$-222(Zn). Samples were measured as nanoparticle suspensions in DMF solvent with λ = 400 nm excitation.

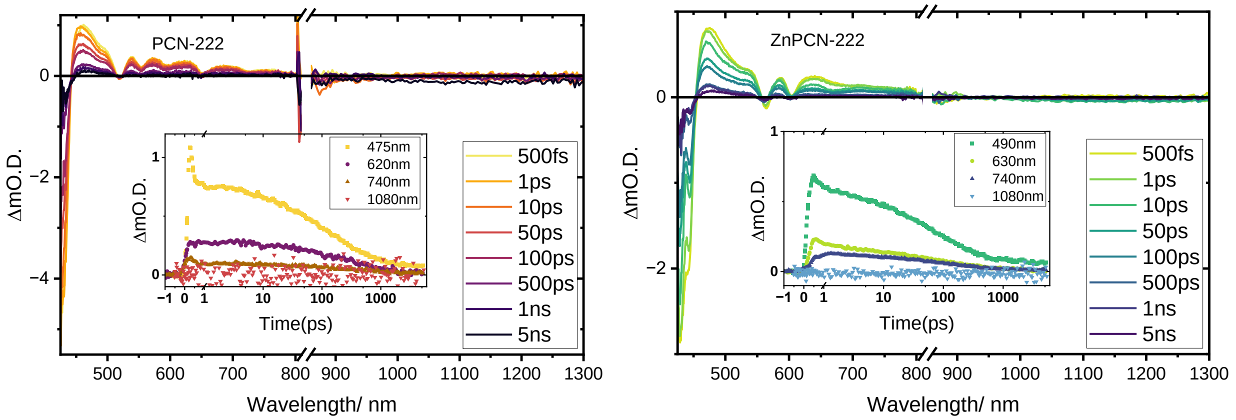

Figure S15 TA spectral overlays of a) PCN-222(H_2) and b) PCN-222(Zn), insets show kinetic slices obtained from the corresponding spectra at 475, 620, 740 and 1080 nm for PCN-222(H_2) and 490, 630, 740 and 1080 nm for PCN-222(Zn). Samples were measured as nanoparticle suspensions in DMF solvent with λ = 400nm excitation.

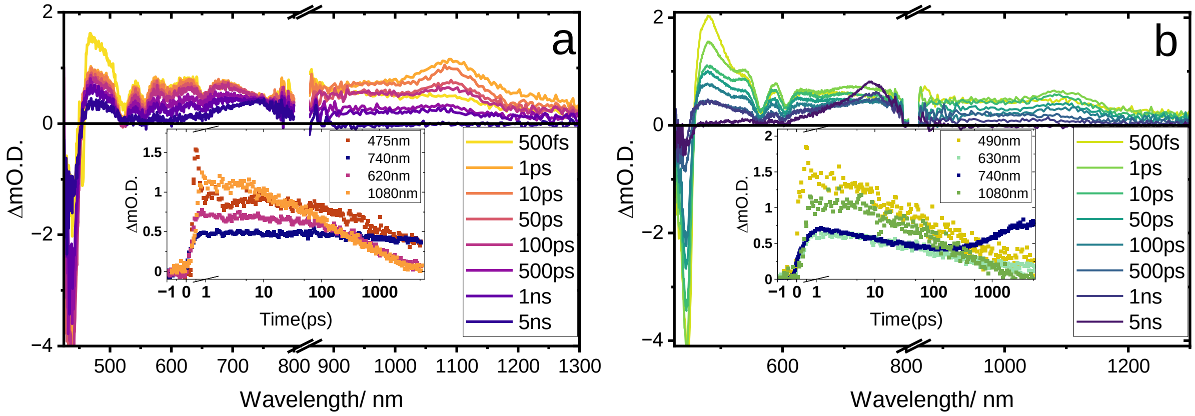

Figure S16 TA spectral overlays of a) C_{60}⊂PCN-222(H_2) and b) C_{60}⊂PCN-222(Zn) in 1,4-dioxane. Insets show kinetic slices obtained from the corresponding spectra at 475, 620, 740 and 1080 nm for C_{60}⊂PCN-222(H_2) and 490, 630, 740 and 1080 nm for C_{60}⊂PCN-222(Zn). Samples were measured as nanoparticle suspensions in 1,4-dioxane solvent with λ = 400nm excitation.

Global Fitting:

Global fitting is performed using a custom code developed in MATLAB. Chirp-corrected and background-subtracted data sets are initially evaluated with singular value decomposition and a parallel decay model to determine the likely number of fit components. The appropriateness of the number of components is gauged by the magnitude and patterns of the residuals. Subsequently, target analysis is performed. The routine is limited to sequential models, including simultaneous sequential models (e.g., A->B->0, C->0). The time constants are initially guessed, and results are checked at multiple initial starting points to arrive at true minima in the fit residuals. The target scheme is judged by how well the resulting spectra match with known features.

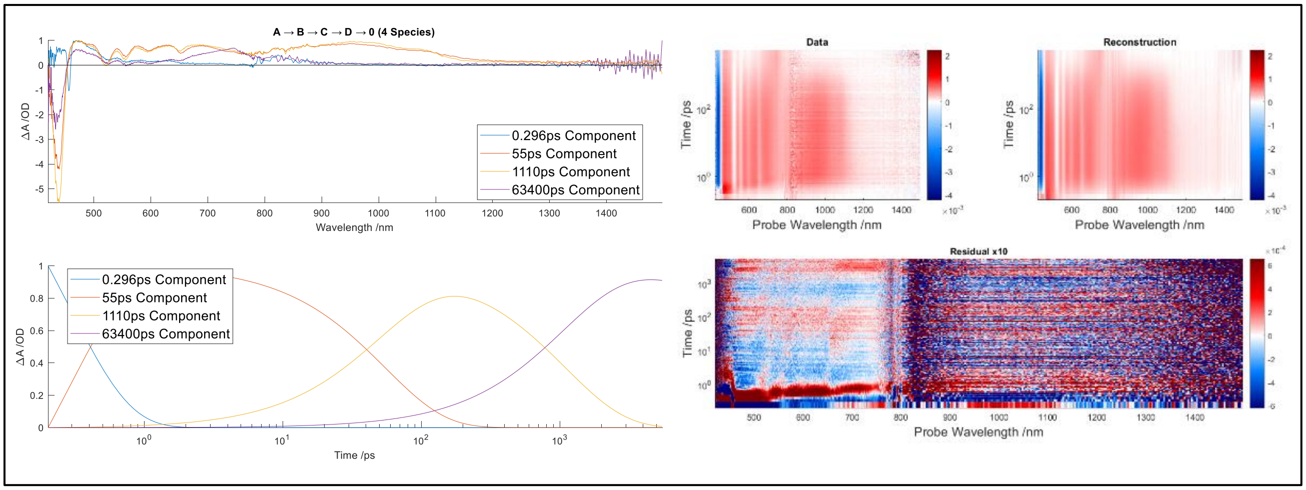

Figure S17 C_{60}⊂PCN-222(H_2) Left) Four-state species-associated spectra retrieved from global analysis of the femtosecond transient absorption of C_{60}⊂PCN-222(H_2) in DMF. A sequential model of A →B → C →D → 0 was applied. Right) The raw fs-TA data (left) along with the reconstructed model (right) and residual of fits (bottom).

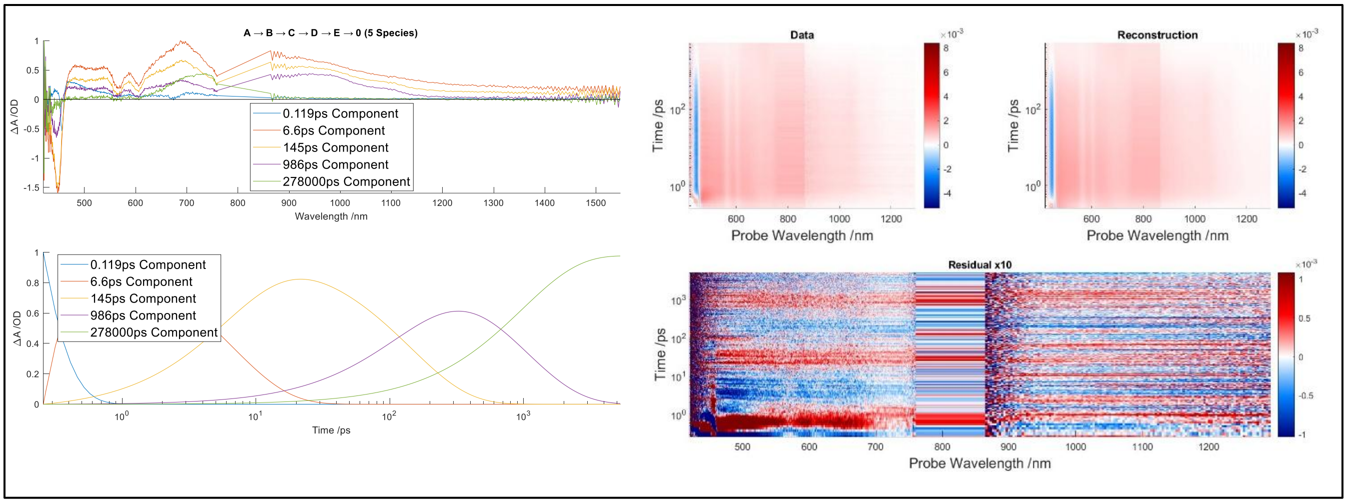

Figure S18 C_{60}⊂PCN-222(Zn) Left) Five-state species-associated spectra retrieved from global analysis of the femtosecond transient absorption of C_{60}⊂PCN-222(Zn) in DMF. A sequential model of A →B → C → D→ E → 0 was applied. Right) The raw fs-TA data (left) along with the reconstructed model (right) and residual of fits (bottom).

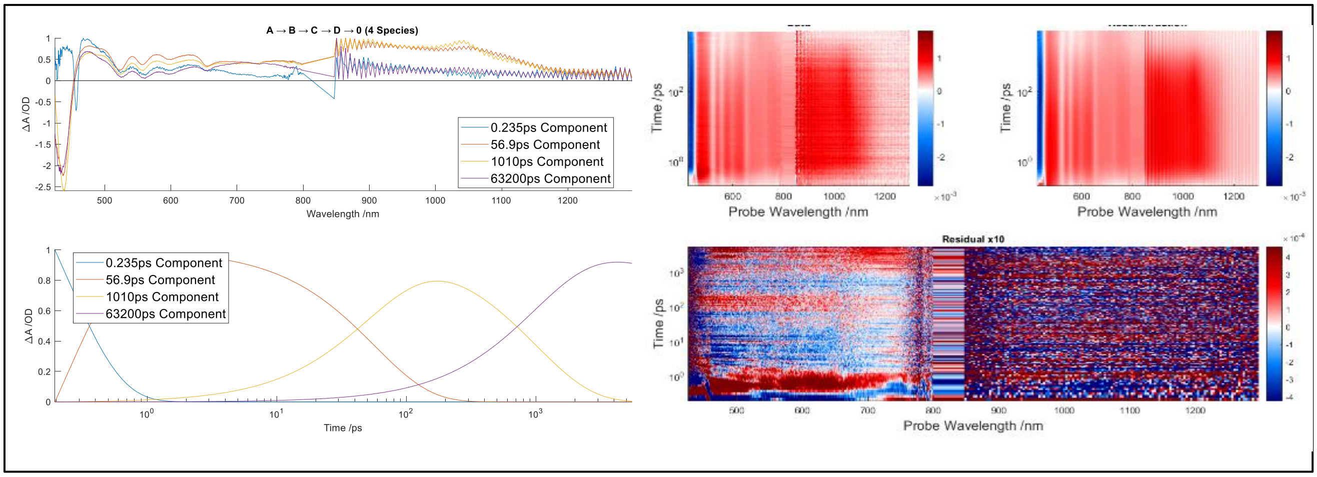

Figure S19 $PC_{61}BM$⊂PCN-222(H_2) Left) Four-state species-associated spectra retrieved from global analysis of the femtosecond transient absorption of $PC_{61}BM$⊂PCN-222(H_2) in DMF. A sequential model of A →B → C → D →0 was applied. Right) The raw fs-TA data (left) along with the reconstructed model (right) and residual of fits (bottom).

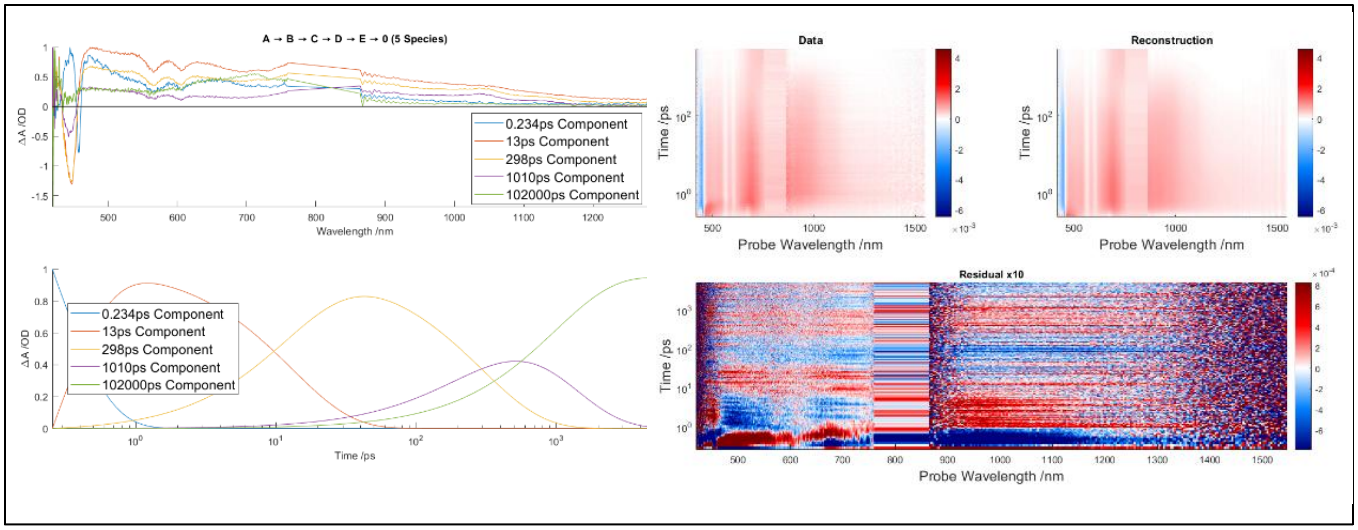

Figure S20 $PC_{61}BM \subset$PCN-222(Zn) Left) Five-state species-associated spectra retrieved from global analysis of the femtosecond transient absorption of $PC_{61}BM \subset$PCN-222(Zn) in DMF. A sequential model of A →B → C → D → E → 0 was applied. Right) The raw fs-TA data (left) along with the reconstructed model (right) and residual of fits (bottom).

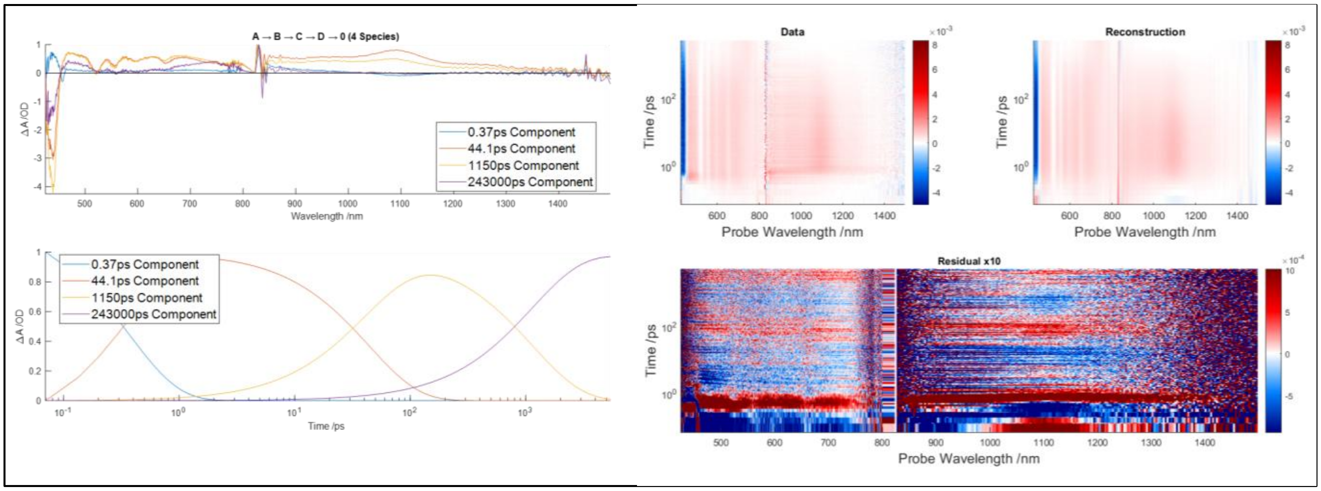

Figure S21 $C_{60} \subset$PCN-222(H_2) in 1,4-dioxane Left) Four-state species-associated spectra retrieved from global analysis of the femtosecond transient absorption of $C_{60} \subset$PCN-222(H_2) in 1,4-dioxane. A sequential model of A →B → C → D → 0 was applied. Right) The raw fs-TA data (left) along with the reconstructed model (right) and residual of fits (bottom).

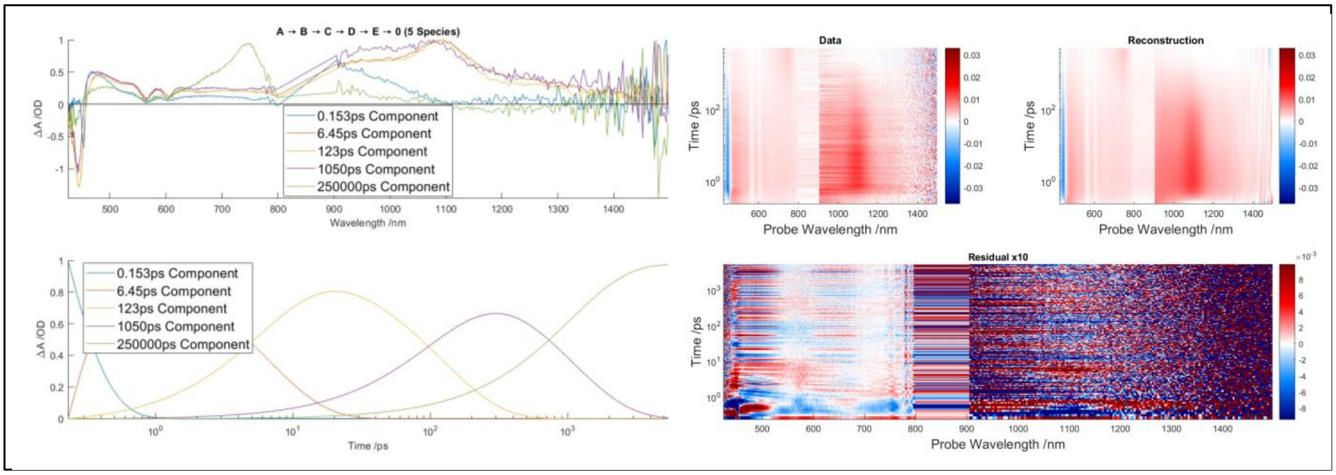

Figure S22 C_{60}⊂PCN-222(Zn) in 1,4-dioxane Left) Five-state species-associated spectra retrieved from global analysis of the femtosecond transient absorption of C_{60}⊂PCN-222(Zn) in 1,4-dioxane. A sequential model of A →B → C → D→ E → 0 was applied. Right) The raw fs-TA data (left) along with the reconstructed model (right) and residual of fits (bottom).

Table S4 Lifetimes of each component extracted from species associated spectra

Sample	S1 → Exciplex (ps)	Exciplex → CT (ps)	CT → Triplet (ps)
C_{60}⊂PCN-222(H_2) (DMF)	0.30 ± 0.05	55 ± 2.48	1110 ± 27.1
$PC_{61}BM$⊂PCN-222(H_2) (DMF)	0.24 ± 0.05	56.9 ± 1.61	1013 ± 21.5
C_{60}⊂PCN-222(H_2) (1,4-dioxane)	0.37 ± 0.05	44.1 ± 1.81	1150 ± 30.3
C_{60}⊂PCN-222(Zn) (DMF)	0.20 ± 0.05	6.6 ± 0.292<br>145 ± 5.88	986 ± 68.2
$PC_{61}BM$⊂PCN-222(Zn) (DMF)	0.23 ± 0.05	13 ± 0.598<br>298 ± 23.1	1008 ± 81.7
C_{60}⊂PCN-222(Zn) (1,4-dioxane)	0.22 ± 0.05	5.95 ± 0.52<br>125 ± 8.84	1060 ± 97.9

Uncertainties reflect fit standard deviations, except for S1 decay, which is limited by the instrument response.

References:

(1) Feng, Dawei; Gu, Zhi-Yuan; Li, Jian-Rong; Jiang, Hai-Long; Wei, Zhangwen; Zhou, Hong-Cai; Zirconium-Metalloporphyrin PCN-222: Mesoporous Metal–Organic Frameworks with Ultrahigh Stability as Biomimetic Catalysts. *Angew. Chem. Int. Ed.* **2012,** *51*, 10307-10310.
